# Position-specific methyl substitution on benzo[a]pyrene drives AHR-dependent fin duplication in zebrafish

**DOI:** 10.1093/toxsci/kfaf164

**Published:** 2025-11-20

**Authors:** Mackenzie L Morshead, Lisa Truong, Robyn L Tanguay

**Affiliations:** Department of Environmental and Molecular Toxicology, Oregon State University, Corvallis, OR 97333, United States; Department of Environmental and Molecular Toxicology, Oregon State University, Corvallis, OR 97333, United States; Department of Environmental and Molecular Toxicology, Oregon State University, Corvallis, OR 97333, United States

**Keywords:** polycyclic aromatic hydrocarbons, transcriptomics, aryl hydrocarbon receptor, cytochrome P450, 8-methylbenzo[a]pyrene

## Abstract

Polycyclic aromatic hydrocarbons (PAHs) are a contaminant class characterized by fused aromatic rings, formed through the incomplete combustion of organic materials and petrogenic sources. Despite the abundance and toxicity of alkyl-substituted PAHs, most research and regulation focus on unsubstituted parent PAHs. Alkyl substitution of Benzo[a]pyrene (BaP), one of the most well-studied parent PAHs, drastically alters its bioactivity in zebrafish. In larval zebrafish exposed from 6 h post-fertilization (hpf), BaP caused behavioral effects but no morphological effects up to 50 µM at 120 hpf. In contrast, 8-methylbenzo[a]pyrene caused a distinct fin duplication phenotype by 0.26 µM and additional morphological effects by 1 µM. Alkyl substitution in different positions (7-, 6-, 9-, and 10-MBaP) did not elicit morphological effects at similar concentrations. This study characterized the morphological effects of 8-MBaP in zebrafish and investigated its mechanism(s) of action. Using knock-out lines, we demonstrated that 8-MBaP toxicity is Ahr2 dependent and that Cyp1a served a protective role. To identify underlying transcriptomic changes, embryos were exposed to 3 concentrations of BaP, 6-MBaP, and 8-MBaP. Whole embryos/larvae were collected at 48 and 72 hpf, which was before and during phenotype onset, respectively. Collecting RNA and morphological effects across concentration, time, and chemicals facilitated the identification of concentration-dependent transcriptional responses linked to the downstream morphological phenotypes unique to BaP methylation at the eighth position. This study improves environmental and human health hazard assessment by identifying critical structural features and mechanisms of action contributing to the toxicity of PAH mixtures in the environment.

Polycyclic aromatic hydrocarbons (PAHs) are present in high concentrations in petrogenic fuels and are produced through the incomplete combustion of organic materials (pyrogenic sources). Benzo[a]pyrene (BaP) is perhaps the most well-studied of the PAHs, a diverse chemical class characterized by fused aromatic rings. BaP, a 5-ring PAH, is often used as a representative for PAH contamination or a reference standard for the carcinogenicity of other PAHs and is highly abundant in pyrogenic sources (Bukowska et al. 2022). A popular hypothesis for the mechanism of BaP carcinogenicity involves aryl hydrocarbon receptor (AHR) agonism, followed by metabolic activation by AHR-induced cytochrome P450 enzymes (CYP1A1 and CYP1B1), formation of benzo(a)pyrene diol epoxide (BPDE), and finally DNA adduction and strand breaks (Bukowska et al. 2022). BaP-induced BPDE DNA adducts are known to disrupt methylation patterns, contributing to intergenerational impacts of BaP exposure (Fang et al. 2013; Bukowska and Sicińska 2021; Pandelides et al. 2023). Another hypothesis suggests that the addition of a methyl group into BaP by methyltransferases forms 6-methylbenzo[a]pyrene (6-MBaP), which in turn is oxidized by AHR-induced cytochrome P450 enzymes to reactive metabolites that cause oxidative damage (Myers et al. 2007; Flesher and Lehner 2016). Beyond carcinogenicity, BaP exposure has been linked to the formation and increased severity of cardiovascular diseases in humans (Fu et al. 2022), zebrafish (Zou et al. 2024), and mice (Kerley-Hamilton et al. 2012). BaP exposure has caused neurodevelopmental effects in mice and abnormal behaviors in larval and adult zebrafish that are transgenerational (Chepelev et al. 2015; Alexiev et al. 2025). Epidemiological studies have associated BaP exposure and the presence of BPDE-DNA adducts with increased rates of cancer, miscarriage, and other developmental and reproductive impacts, including neurodevelopmental delays and DNA damage in gametes (Zenzes 2000; Perera et al. 2005; Amadou et al. 2021; Tartaglione et al. 2023; Guo et al. 2024).

Due to the ubiquitous nature of combustion pollution, human exposure to BaP is widespread and well documented. The primary routes of exposure to BaP are inhalation of polluted air and cigarette smoke and the ingestion of contaminated foods, especially those exposed to high-temperature processes (Bukowska et al. 2022). Although the environmental occurrence of BaP is well studied, other major contributors to PAH mixtures are not, especially substituted derivatives including alkylated derivates (Golzadeh et al. 2021). Despite evidence of their abundance, alkylated PAHs are not part of routine air and water quality monitoring. When measured, they are repeatedly found in higher concentrations than parent PAHs in air, water, soil, and traditional food sources (Qiao et al. 2020; Golzadeh et al. 2021; An et al. 2022; Grung et al. 2022; Moradi et al. 2022; Qian et al. 2022). There are minimal environmental sampling data available for alkylated BaPs specifically, with most studies focused on 6- and 7-MBaP detected at the same or higher concentration than BaP in mining and coal field sites (An et al. 2022; QiAn et al. 2022).

Studies of the biological activity of alkylated BaPs are largely limited to the mutagenic potential of monomethyl-substituted BaPs. This substitution can occur at 12 positions, 1-MBaP to 12-MBaP. A summary of previously reported mutagenic potential and tumor-initiating activity of BaP and its 12 monomethyl-substituted derivatives is reported in Wang et al. (2022). In summary, these studies found that the mutagenicity increased or decreased based on methyl group position. Activity trends observed in the Ames assay were variable between strain and study and were not reflected in a tumorigenesis study (Iyer et al. 1980). Wang et al. (2022) further assessed the mutagenicity of Bap, 3-, 6-, 7-, and 8-MBaP, which varied with the bacterial strain used for the Ames assay, with 6-MBaP being most potent in TA98 and 3-MBaP being most potent in TA100. In vitro metabolic oxidation of alkylated BaPs favors the aliphatic side chain, shifting oxidation away from the aromatic ring (Wang et al. 2022). This is especially true for 3-, 6-, and 8-MBaP, which have increased steric hindrance for aromatic ring oxidation (Wang et al. 2022). Aromatic ring oxidation is necessary for the formation of dihydrodiol-epoxide metabolites, associated with BaP-induced DNA damage. Thus, alkylated BaPs that produce minimal ring oxidation metabolites likely act through a different metabolic mode of action than BaP or are not acting through metabolic activation (Wang et al. 2022).

Although there are established impacts of BaP exposure on developmental endpoints, developmental toxicity studies of alkylated BaPs are limited. Previously, we studied the developmental toxicity of 104 alkylated PAHs derived from 11 parent compounds. Of all the alkylated PAHs, 8-MBaP was the most potent (Morshead et al. 2025). The contrast between 8-MBaP and the other 5 alkylated BaPs (7-, 8-, 9-, and 10-MBaP and 7,10-dimethylbenzo[a]pyrene) was striking. Although all induced *cyp1a* expression, 8-MBaP was the only one that caused morphological effects. The most potent effect was a lower trunk abnormality, also referred to as caudal fin duplication (x-fin), at a benchmark concentration (BMC) of 50% effect above background (BMC_50_) of 0.3 µM (Shankar et al. 2019; Morshead et al. 2025). There are likely other developmental-specific mechanisms besides DNA damage and oxidative stress that led to the x-fin phenotype.

This study aims to characterize the unique and potent morphological effects of 8-MBaP in zebrafish and identify the underlying mechanism(s) of action. To do this, we compared the effects of another monomethyl-substituted BaP (6-MBaP) and BaP to those of 8-MBaP. 6-MBaP was chosen as the monomethyl-substituted BaP for comparison due to its mutagenic potential indicated by the Ames assay and implication in the bioalkylation hypothesis of BaP toxicity (Myers et al. 2007; Flesher and Lehner 2016; Wang et al. 2022). Knock-out (KO) lines of the 3 zebrafish AHR proteins and Cyp1a were used to identify the dependence of the 8-MBaP phenotype on each of these proteins individually. Lastly, concentration–response RNA sequencing data were collected from embryos/larvae prior to and at the emergence of the 8-MBaP phenotype. Collecting RNA and morphological effects across concentration, time, and chemical facilitated the identification of concentration-dependent transcriptional responses linked to downstream morphological phenotypes unique to BaP methylation at the eighth position. We compared our results to transcriptomic data from 2 other chemicals that cause the x-fin phenotype to identify common differentially expressed genes. Our identification of position-specific effects of BaP methyl substitution and the underlying toxic mechanisms is critical to understanding the hazards posed by the abundant but often overlooked alkylated PAHs.

## Materials and methods

### Zebrafish husbandry and chemical exposure

Adult zebrafish (*Danio rerio*) used in this study were maintained under Institutional Animal Care and Use Committee protocol 2021-0227 at Oregon State University, Sinnhuber Aquatic Research Laboratory (SARL, Corvallis, OR). Adult fish were fed Sparos Zebrafeed 300 µM twice per day, kept in densities of ∼500/35-gallon tank (5D) and 3-12/l in 6-l tanks (KO lines), in a 14:10 light:dark cycle. Recirculating treated water filtered through 10 µm, activated charcoal, and UV, was prepared using reverse osmosis water supplemented with Instant Ocean salts and sodium bicarbonate to achieve a conductivity of 1,000 µS and a pH of 7.4. For 5D fish, a specialized gridded platform and funnel were placed in the mass spawn tank the night before, and spawning was initiated at lights on at 8 AM. For the KO line, small group spawning was conducted by placing fish in spawning baskets the night before, and spawning was initiated at lights on at 8 AM. Embryos were collected and screened to exclude unfertilized and abnormals, then sorted to uniform developmental stages (Kimmel et al. 1995). Chorions were enzymatically removed at 4 h post-fertilization (hpf) for all experiments except KO line exposures due to low spawn yields (Mandrell et al. 2012). At 6 hpf, plastic 96-well round-bottom tissue culture-treated plates were prefilled with 100 µL of embryo medium (EM), and single embryos were loaded into each well using our automated embryo placement system (Mandrell et al. 2012). Embryo quality was checked 30 min after plating, and damaged or malformed embryos were replaced. At 7 hpf, embryos were exposed to chemical stocks dispensed into 96-well plates using the Hewlett-Packard D300e digital dispenser and normalized to 1% dimethyl sulfoxide (DMSO), previously identified not to cause developmental toxicity (Truong et al. 2016; Hoyberghs et al. 2021). Plates were kept for 18 h on a shaker at 225 revolutions per minute at 28 °C in the dark until 24 hpf, when embryos were assessed for mortality, then kept stationary in the dark at 28 °C until larvae were assessed for 120 hpf morphology. Solutions were not exchanged during the experiment.

### Chemicals

Analytical grade stock solutions of the chemicals used in this study (8-methylbenzo[a]pyrene (8-MBaP), 6-methylbenzo[a]pyrene (6-MBaP), and benzo[a]pyrene (BaP)) were obtained from and verified by the Chemical Mixtures Core of the Oregon State University Superfund Center. Standards were purchased neat and dissolved in DMSO. Table 1 contains the CAS number, vendor, purity, and stock solution concentration for each chemical.

**Table 1.** Analytically verified chemical stock information.

Chemical name	CAS number	Vendor	Purity	Stock conc. (mM)	Structure
8-methylbenzo[a]pyrene (8-MBaP)	63041-76-9	Alfa chemistry	99.60%	1.33	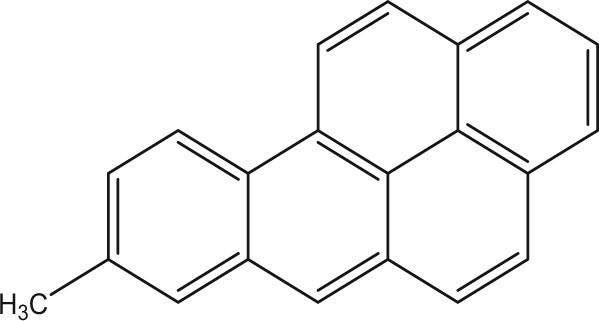
6-methylbenzo[a]pyrene (6-MBaP)	2381-39-7	Beantown Chemical	96%	5.22	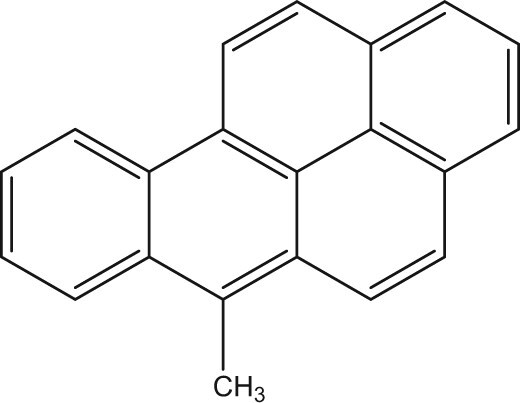
benzo[a]pyrene (BaP)	50-32-8	Accustandard	94.60%	5.03	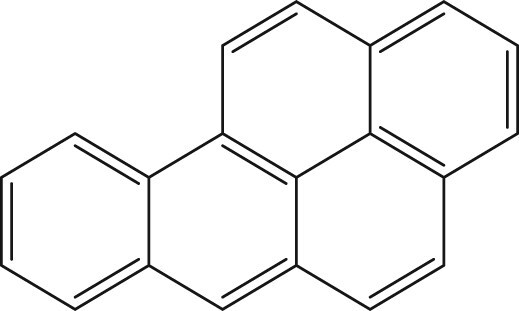

### Morphology concentration response

Chemical exposure for morphological assessments was conducted over 2 identical 96-well plates with 12 concentrations and controls, resulting in *n* = 14 for each concentration and *n* = 24 for controls. 6-MBaP and BaP concentrations ranged from 1 to 15 µM in a linear series, and 8-MBaP concentrations ranged from 0.1 to 13.3 µM in a logarithmic series. Maximum concentrations for 6-MBaP and BaP were determined based on solubility limits. The maximum concentration of 15 µM was the highest exposure concentration before chemical precipitate was visible in the wells. The maximum concentration of 13.3 µM used for 8-MBaP was the highest achievable concentration due to stock concentrations and the 1% DMSO limit, which did not cause morphological effects but fostered solubility (Hoyberghs et al. 2021).

At 120 hpf, larvae were screened for the presence or absence of morphological abnormalities binned into 10 endpoints: Mortality, cranial, axis, edema, muscular/cardiovascular, lower trunk, brain, skin/pigmentation, notochord, and x-fin. These endpoints are described more thoroughly in the online supplementary material T1. Morphological assessments were made by a team of 3 to 4 individuals. .  Representative images of the caudal fin malformation (x-fin) phenotype are included in Fig. 2A and Fig. S1. The incidence of 5 endpoints (x-fin, edema, cranial, axis, and mortality) was above background levels; thus, only these were discussed. The incidence of all endpoints is reported in online supplementary material T2.

#### Statistics

BMC response values were estimated as previously described (Truong et al. 2022). Briefly, concentration and response data were fit to a 3-parameter log-logistic model following US Environmental Protection Agency (US EPA) guidelines described in BMDS v3.2 (US Environmental Protection Agency [EPA] 2020). The 3 model parameters were identified using maximum likelihood estimation. These models were used to compute the BMC for a 20% response (BMC_20_) for each endpoint using “extra risk.”

### *Cyp1a* reporter line expression

The induction of cytochrome P450 enzyme expression by AHR agonism is implicated in the bioactivation of benzo[a]pyrene and its methylated derivatives. Cytochrome P450 1A (CYP1A) is highly responsive to AHR agonism and is thus an excellent biomarker for AHR agonism. We used a *cyp1a* reporter line (*Tg*[*cyp*1*a: Nls*-*egfp*]) to characterize and compare the AHR agonistic activity of all 3 chemicals. This line expresses green fluorescence protein under the endogenous zebrafish *cyp1a* promoter. Embryos were exposed to each chemical at 1.33 µM (*n* = 24); this concentration resulted in larvae at 120 hpf with sufficiently normal morphology for imaging for all chemicals. Sixteen fish with sufficiently normal morphology were randomly selected and loaded into a flat glass-bottom 96-well plate with 50 µl of EM and 5 µl of tricaine. All wells were imaged using the Oxford Andor BC43 confocal microscope at 2× using a 529 nm laser set at 10 and 200 ms exposure. Four representative fish from each exposure were selected for mounting in 1% low-melt agarose and imaging at 10× using the same acquisition settings for a 100-step Z-stack with auto step-size over the width of the larvae. Some organs are visible only from the right or left of the fish. To capture expression in all organs, 2 fish were imaged on their right and 2 on their left for each treatment.

A stitched overview image of 16 larvae from each treatment imaged at 2× in a 96-well plate format, with identical visualization dynamic range parameters, is available in Fig. S2. Representative fish from each treatment were imaged to better visualize differences in spatial expression profiles of each chemical exposure; image acquisition parameters at 10× magnification were kept the same, but visualization dynamic range was set to 150 to 500 for BaP and 6-MBaP (Fig. S3) and 150 to 7,000 for 8-MBaP.

### 8-Methylbenzo[a]pyrene KO line exposures

KO line exposures were conducted to investigate the AHR and Cyp1a dependence of the 8-MBaP-induced x-fin phenotype. We used lines functionally null for Ahr1a (*ahr1a^osu6^*) (Morshead et al. 2025), Ahr1b (*ahr1b^wh36^*) (Karchner et al. 2017), Ahr2 (*ahr2^hu3335^*) (Goodale et al. 2012), and Cyp1a (*cyp1a^osu4^*) (Rude et al. 2024). Two 96-well plates of each line were exposed to 8-MBaP using the same experimental design as previously described in the section “Morphology concentration response.” Due to limitations in spawning capacity, the chorions were not enzymatically removed in these exposures. Morphological assessments were conducted at 120 hpf, and BMC_20_ concentrations were determined for each endpoint as described in the section “Morphology concentration response.” Morphological percent effect data are available in online supplementary material T3.

### 8-Methylbenzo[a]pyrene phenotype time course

A time course experiment was conducted to assess the emergence of the morphological effects following 8-MBaP exposure. Embryos were exposed to 3 concentrations of 8-MBaP (0, 0.133, 1.33, and 13.3 µM, *n* = 24). Morphological assessment for mortality was performed at 24 hpf, and full morphological assessment for the endpoints discussed in “Morphology concentration response” section was performed at 48, 60, 72, 96, and 120 hpf. Morphological percent effect data are available in online supplementary material T4. A Fisher’s exact test with a Holm’s multiple comparison correction was used to test statistical significance for each phenotype at each concentration and timepoint. An adjusted *P*-value of ≤0.05 was considered significant.

### Transcriptomics

Poly-A tail bulk RNA sequencing was performed for 3 chemicals (8-MBaP, 6-MBaP, and BaP) at 3 concentrations and 2 timepoints to elucidate transcriptomic changes unique to 8-MBaP exposure and development of the x-fin phenotype.

#### Exposures and RNA collection

All chemicals were exposed at the same concentrations (0, 0.133, 1.33, and 13.3 µM). These concentrations were phenotypically anchored to 8-MBaP morphological phenotypes. Concentrations were picked that cause no morphological effects (0.133 µM), only x-fin (1.33 µM), and x-fin as well as cranial malformations and edema (13.3 µM). Timepoints were chosen to be before the observation of any morphological effects (48 hpf) and at the onset of morphological effects (72 hpf).

For each chemical and concentration combination, a 96-well plate was exposed, as well as 1 plate of control embryos, following the methods described in the “Morphology concentration response” section. One plate for each chemicalwas concurrently exposed and treated identically to plates used for transcriptomic sample collection with *n* = 24 for each concentration and control to assess morphological effects at 120 hpf; morphological percent effect data are available in online supplementary material T5. At 48 and 72 hpf, living embryos/larvae were collected at random regardless of morphological phenotypes and pooled in groups of 6. Five replicates of each chemical and concentration combination and control were collected at each timepoint, and the pooled sample EM volume was normalized to 75 µl. Collection plates were placed on ice for 1 min, immediately followed by the addition of 100 µl of RNA shield. Plates were sealed with foil seals and kept at −80 °C until RNA extraction.

#### RNA extraction and QC

RNA extraction was performed using the KingFisher Apex System (ThermoFisher Scientific, Waltham, MA, United States) and *Quick*-RNA Magbead kit (Zymo Research Corporation, Irvine, CA, United States). Samples were thawed and combined into the homogenization plate (2 ml deep 96-well plate pre-filled with 400 µl 1 mm zirconium silicate beads and 525 µl of lysis buffer) and sealed with a silicone seal. Using the Mini-BeadBeater 96 (BioSpec Products, Bartlesville, OK, United States), the plate was homogenized for 1.5 min and centrifuged for 5 min at 3000 rcf; 550 µl of the supernatant was distributed over 2 plates, each pre-filled with 275 µl of ethanol. Extraction was an automated process that used Mag beads to collect RNA, after a series of washes and treatment with DNase, the RNA is eluted in 50 µl of ultra-pure water. RNA quality was assessed using the RNA ScreenTape and the 4200 TapeStation System (Agilent, Santa Clara, CA, United States). The RNA purity was assessed using the 260/230 ratio measured with the Varioskan ALF multimode plate reader (ThermoFisher Scientific, Waltham, MA, United States). RNA concentration was determined using Quant-iT broad-spectrum RNA Reagent and assay kit and Varioskan ALF multimode plate reader (standard curve *R*^2^ = 0.996). The 4 replicates of each treatment with the highest RNA concentration, which met RNA quality and purity criteria (2.3 ≥ 260/230 ≥ 1.8, RIN = 10), were selected for sequencing. The resulting 80 samples were normalized using ultrapure water to the lowest concentration, 56 ng/µl.

#### Sequencing

RNA sequencing was performed by Alithia Genomics (Epalinges, Switzerland) using their Mercurius BRB-SEQ service, which is a multiplexed 3′ RNA sequencing library preparation technique. Samples were sequenced using Element Biosciences AVITI sequencer with ∼9.6 million reads/sample. Reads were mapped and demultiplexed by Alithia Genomics; 88% of reads were mapped to the genome, and 67% were mapped to exons.

#### Data analysis

Further processing of read count data was performed using R version 4.4.2. Principal component analysis of log_2_ counts per million (CPM) read counts showed that samples were clustered based on timepoint due to developmental differences in mRNA expression (Fig. S4). Further analysis was performed on the samples from each time-point separately.

Differential expression analysis was conducted on read counts filtered for ≥5 reads for at least 2 samples using DeSeq2; treatment groups were compared with their respective time-point controls (Love et al. 2014). Genes were considered differentially expressed if adjusted *P*-values ≤0.05 with no log_2_ fold change cut off.

##### Concentration response modeling

Data were filtered for low read counts and normalized, then split into chemical groups for each time-point and further filtered to include only genes that were differentially expressed in at least 1 concentration for concentration response modeling using ExpressAnalyst (www.expressanalyst.ca). Raw read counts were filtered to include only genes with ≥5 reads for at least 2 samples, resulting in a total of 17,794 and 18,432 genes at 24 and 48 hpf, respectively. Normalization to CPM was performed using the calcNormFactors function, then log_2_ transformed (log_2_CPM) using the edgeR package (Robinson et al. 2010). Log_2_CMP values for each chemical and timepoint were filtered to include only genes that were differentially expressed in at least 1 concentration. These values were used as input for concentration–response modeling using FastBMD incorporated into ExpressAnalyst (Ewald et al. 2021). FastBMD implements recommendations for genomic dose–response modeling following National Toxicology Program’s (NTP) 2018 recommendations. No filtering, gene annotation, or transformation was applied within ExpressAnalyst, and all genes within each filtered chemical and timepoint grouping were fit to 6 models (Exp2-4, Linear, Poly2, and Power). The best-performing model was identified using the Akaike information criterion, and the best-fit model was used to calculate the BMC for each gene. The BMC is the estimated concentration that produces a predetermined change in the gene expression or the benchmark response (BMR). The BMR for each gene was set at the default of 1.35 standard deviations from control expression. Model fitting results for genes for which ExpressAnalyst was able to calculate a BMC within the concentration range and a 95% confidence interval BMC upper (BMCu) and BMC lower (BMCl) are available in online supplementary material T8. These genes were further filtered to include only those with a BMCu/BMCl ≤40. Genes meeting these criteria were considered to have a concentration response relationship; however, genes with the best-fit model Poly2 were removed for further analysis due to their non-monotonic responses. As the morphological phenotypes of interest in this study have a monotonic response, we determined that exclusion of non-monotonic responding genes would improve clarity in mRNA sequencing analysis. Genes with BMC values ≤1.33 µM for 8-MBaP were considered x-fin-associated genes, as they were modeled to be disrupted at or below 1.33 µM (the lowest tested concentration in transcriptomic analysis to cause x-fin). In contrast to simply selecting DEGs at 1.33 µM 8-MBaP, this concentration response approach captured genes that were not yet differentially expressed by 1.33 µM, as well as filtered out genes that may have been differentially expressed at 1.33 µM but did not have a monotonic concentration response.

##### Functional enrichment analysis

Functional enrichment analysis using gene ontology biological process (GO:BP) terms was performed using the ClueGo plug-in for Cytoscape (Shannon et al. 2003). Details on the functional enrichment analysis performed by ClueGo are available in Bindea et al. (2009). Briefly, ClueGo creates a network visualization of functional groups enriched in the gene list provided. The functional term similarity is calculated using chance-corrected kappa statistics to determine the strength of the association between terms. The network represents each significant term for the gene set as nodes, and edges between nodes are drawn based on a predefined kappa score level ≥0.35 and a percent gene overlap of at least 50%. Functional enrichment networks were created for x-fin-associated genes at 48 hpf and 72 hpf. The names of all enriched GO:BP terms and *P*-values associated with the enrichment, as well as gene lists for each term, are available in online supplementary material T9.

##### Comparison with previous data

X-fin-associated genes at 48 hpf, before the emergence of the phenotype, were filtered to exclude genes with a concentration response in either BaP or 6-MBaP (x-fin unique genes). X-fin unique genes were compared against genes differentially expressed at 48 hpf following exposure to benzo[*j*]fluoranthene (BjF) or benzo[*k*]fluoranthene (BkF), previously published by Shankar et al. (2019), using the same 96-well plate method described in this study. Both BjF and BkF exhibit the same x-fin phenotype described in the current study. Embryos collected for RNA sequencing were exposed to concentrations that cause x-fin (referred to as caudal fin malformation) in 80% of larvae at 120 hpf, 14.9 µM and 1.9 µM for BjF and BkF, respectively. Pools of 8 embryos were collected at 48 hpf for sequencing. Differential expression analysis was conducted using DeSeq2; genes with an adjusted *P*-value ≤0.05 were considered differentially expressed. Further experimental details can be found in Shankar et al. (2019). Results from the differential expression analysis for BjF and BkF used in this study are available in online supplementary materials T10 and T11, respectively.

X-fin unique genes differentially expressed in both BjF and BkF exposures were compared against sequencing of fin tissue of embryos exposed to 12 µM BkF, previously published by Garland et al. (2020), using the same 96-well plate method described in this study. RNA sequencing was performed on caudal fin tissue pooled from 50 embryos collected at 48 hpf. Differential expression analysis was performed using DeSeq2; genes with an adjusted *P*-value ≤0.05 were considered differentially expressed. Further experimental details can be found in Garland et al. (2020). Results from the differential expression analysis for BkF fin tissue used in this study are available in online supplementary material T12.

## Results and discussion

### 8-Methybenzo[a]pyrene disrupted caudal fin formation

**Fig. 1.**
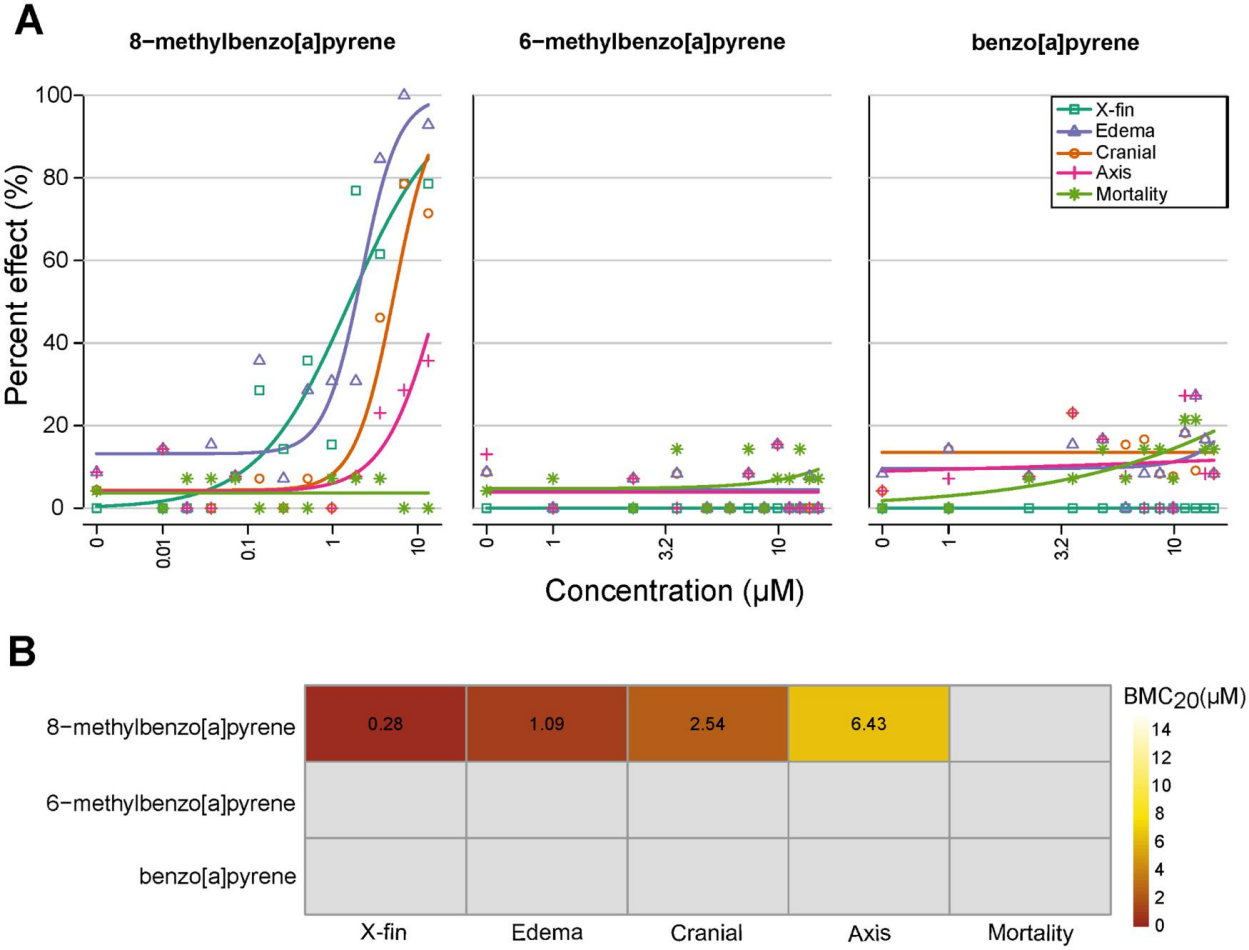
Morphological effects of embryonic exposure to 8-methylbenzo[a]pyrene, 6-methylbenzo[a]pyrene, and benzo[a]pyrene. Exposures from 7 to 120 h post-fertilization (hpf). A) Concentration response curves for each endpoint using a 3-parameter log-logistic model. B) Heatmap displaying the benchmark concentration at 20% effect (BMC_20_) for each endpoint: chemical combination.

**Fig. 2.**
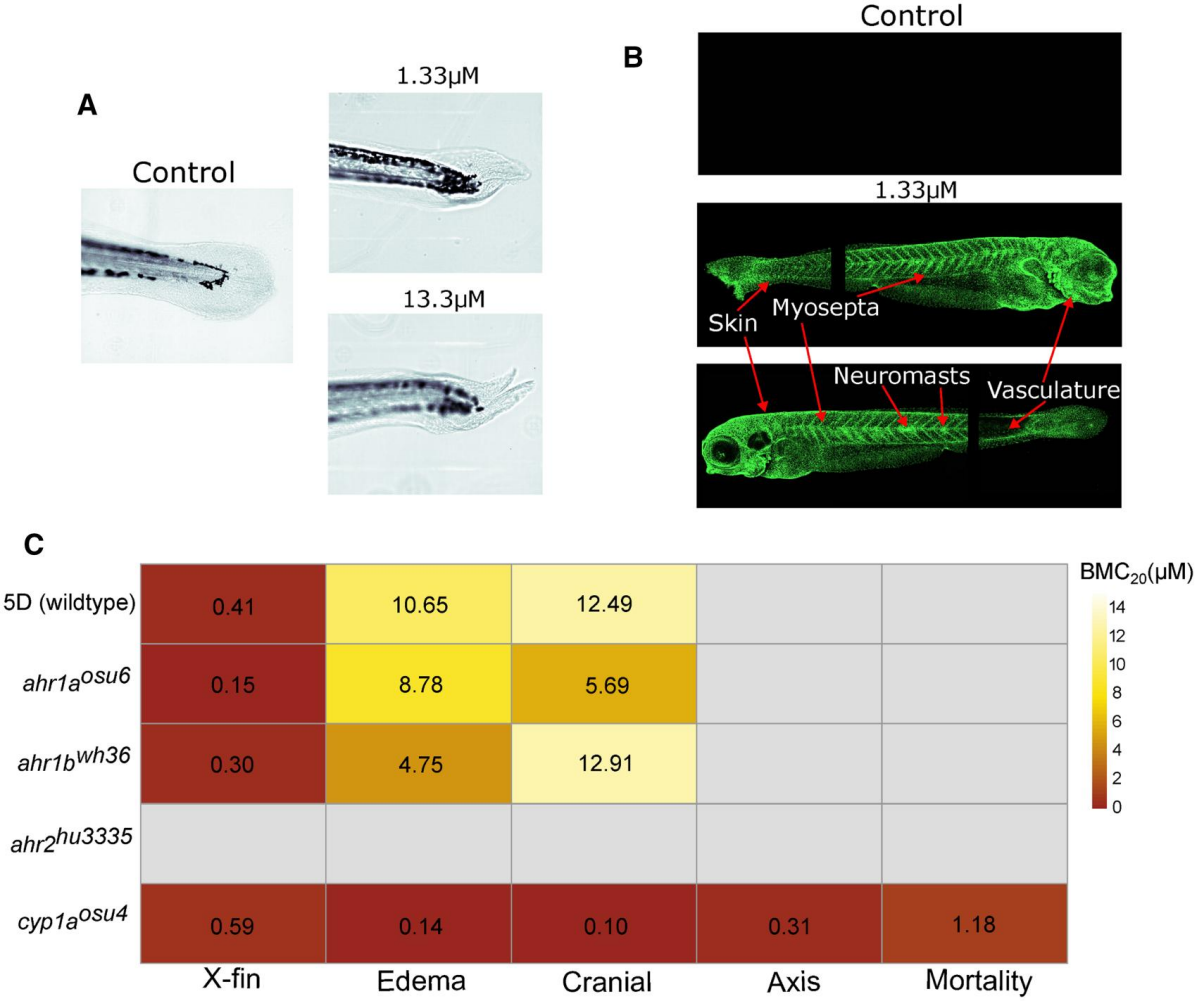
A) Representative images of x-fin phenotype at 120 hpf following embryonic exposure to 8-methylbenzo[a]pyrene at 1.33 and 13.3 µM. B) Lateral fluorescence images of a *cyp1a* reporter line (*Tg*[*cyp*1*a: Nls*-*egfp*]) at 120 hpf following embryonic exposure to 1.33 µM 8-methylbenzo[a]pyrene imaged from both right (top) and left (bottom). The section “Statistics” under “Morphology concentration response” discusses the rationale for imaging on both sides. Locations of expression are indicated with red arrows. C) BMC_20_ values for each effected morphological endpoint following embryonic exposure to 8-methylbenzo[a]pyrene in wildtype (5D) and knock-out lines for Ahr1a (*ahr1a^osu6^*), Ahr1b (*ahr1b^wh36^*), Ahr2 (*ahr2^hu3335^*), and Cyp1a (*cyp1a^osu4^*).

8-MBaP caused potent morphological effects, whereas exposure to 6-MBaP and BaP did not result in significant effects. Our morphological assessment confirmed the previously reported lower trunk abnormality due to 8-MBaP exposure (Morshead et al. 2025). The lower trunk abnormality was consistent with the development of an ectopic caudal fin fold perpendicular to the regular caudal fin fold, previously described by Garland et al. (2020); representative images are shown in Fig. 2A (additional representative images are shown in Fig. S1). This phenotype, referred to as “x-fin,” was associated with 4 other PAHs (dibenzo[*b, k*]fluoranthene, dibenzo[*a.h*]anthracene, BjF, and BkF) (Geier et al. 2018; Garland et al. 2020). Of the PAHs previously reported to cause the x-fin, BkF was the most potent, with a lowest effect level (LEL) of 1 µM for x-fin and 35.5 µM for edema (Shankar et al. 2019). Other x-fin-inducing chemicals did not cause additional morphological effects. 8-MBaP was both more potent in causing the x-fin phenotype (BMC_20_ value of 0.28 µM), produced edema at lower concentrations than BkF, and produced additional cranial malformations and axis abnormalities at higher concentrations (Fig. 1).

A previous study by Fang et al. (2022) identified morphological effects of 8-methylbenzo[a]pyrene in an embryonic zebrafish exposure; however, the most potent effect in their study was no hatch, followed by edemas and protruding jaw. There was no description of caudal fin malformation; however, the no-hatch phenotype may make identification of caudal fin malformations difficult. The authors observed morphological effects from BaP exposure, but no effect on hatching. Several differences from the present study may explain the discord. Fang et al. (2022) exposed chorion-intact embryos in 2 ml of medium up to 50 µM using 0.25% DMSO, assessing for morphological effects at 96 hpf. The current study exposed dechorionated embryos in 100 µl of medium up to 13.3 µM using 1% DMSO, assessing for mortality at 24 hpf and mortality and morphological effects at 120 hpf. Using 2 ml of exposure volume increased the available chemical mass/embryo ratio by 20× compared with our study. We observed that aqueous exposure of BaP and its alkylated derivatives above 15 µM were not completely soluble; this, in combination with the lower percent DMSO used by Fang et al. (2022), could cause solubility issues in their study (Kwon and Kwon 2012). It is possible that non-solubilized chemical acts as a replenishing source as the soluble chemical is metabolized (Kwon and Kwon 2012; Rude et al. 2025).

The morphological effects of 8-MBaP contrasted the lack of effects from 6-MBaP and BaP, highlighting the impact that small structural changes can have on the biological effects of chemical exposure. Both 6-MBaP and BaP were included in this study due to their reported mutagenic potential and, in the case of BaP, other developmental effects. However, neither of these chemicals elicited morphological effects at the concentrations tested. BaP has been tested previously for developmental toxicity in zebrafish. Many studies have found notable impacts on larval behavior from embryonic exposure to BaP as well as reproductive and cardiovascular effects in adults following developmental BaP exposure (Gao et al. 2018; Geier et al. 2018; Hawkey et al. 2022; Tartaglione et al. 2023). However, morphological effects in larvae following embryonic exposure are inconsistently reported, likely because of differences in experimental design (Incardona et al. 2011; Geier et al. 2018; Elfawy et al. 2021; Fang et al. 2022). The observed developmental effects of BaP have been attributed to oxidative stress-induced apoptosis (Elfawy et al. 2021). 6-MBaP, however, has not been previously assessed for developmental toxicity; the lack of morphological effects is consistent with previously screened, singly alkylated BaPs, including 7-MBaP tested up to 100 µM, and 9- and 10-MBaP tested up to 1.9 µM (Morshead et al. 2025). Among the alkylated BaP derivatives tested, only substitution at the eighth position appears to cause teratogenicity.

### 8-Methylbenzo[a]pyrene fin duplication is AHR dependent and Cyp1a independent

Whether AHR binding, increases chemical elimination, promotes the formation of toxic metabolites, or causes transcriptomic changes that disrupt development, it is well established that many PAHs, including BaP, are potent ligands for the AHR. The induction of CYP1A is often used as a proxy for AHR agonism, and a previous screen identified that 8-MBaP can induce a *cyp1a* expression (Morshead et al. 2025). Based on this and the findings of Garland et al (2020), which showed the Ahr2 dependence of BkF-induced x-fin, we suspected that the AHR might also play a role in the 8-MBaP-induced x-fin phenotype (Garland et al. 2020).

The spatial induction of *cyp1a* was assessed using a cyp1a-GFP reporter line. Larvae were exposed at 6 hpf to 8-MBaP, 6-MBaP, or BaP at 1.33 µM and imaged at 120 hpf. The induction of *cyp1a* by 6-MBaP and BaP was indistinguishable from that of the control larvae at the exposure length, laser intensity, and visualization dynamic range satisfactory for capturing the fluorescence of 8-MBaP-exposed *cyp1a* reporter larvae (Fig. S2). *Cyp1a* expression in 8-MBaP exposure was significantly different from the control larvae and highest in the skin, but expression in the neuromasts, vasculature, and myosepta was also present (Fig. 2B). Although the intensity of *cyp1a* induction by exposure to 6-MBaP and BaP was significantly lower than the induction by 8-MBaP, the expression locations were the same, with BaP induction slightly stronger than 6-MBaP induction (Fig. S3). This difference in AHR affinity or unique ligand-specific impacts of AHR binding likely played a role in the morphological effects from exposure to 8-MBaP.

The dependence of the 8-MBaP phenotype on the AHR and Cyp1a was assessed using KO lines. As zebrafish express 3 AHR proteins due to genome duplication events in their evolutionary history, 3 KO lines were used: *ahr1a^osu6^*, *ahr1b^wh36^*, and *ahr2^hu3335^* (the functional analog to mammalian AHR). These and a Cyp1a KO line (*cyp1a^osu4^)* were exposed to a broad concentration range of 8-MBaP. Embryos from the transgenic lines were left in their chorions due to low spawning yields, which had no impact on the x-fin BMC_20_ but did increase the BMC_20_ for both edema and cranial malformations and reduced effects on the axis at 120 hpf. In contrast to the findings of Fang et al. (2022), there were no impacts from 8-MBaP exposure on hatch rate in any of the lines, including 5D (wildtype) (Fang et al. 2022).

Although knocking out *ahr1a* or *ahr1b* had no impact on the morphological effects of 8-MBaP, knocking out *ahr2* eliminated the morphological effects, including x-fin formation (Fig. 2C). Interestingly, knocking out Cyp1a did not affect the x-fin BMC_20_ but decreased it for edema and cranial effects and caused emergent effects, including curved axis and mortality at low concentrations (BMC_20_ 0.31 and 1.18 µM, respectively) (Fig. 2C). Concentration-response curves for each endpoint compared across lines showed that the x-fin phenotype for the Cyp1a KO line did not reach the same maximal effect level as wild type (5D) due to increases in mortality (Fig. S5). Based on these findings, we conclude that the morphological effects of 8-MBaP require the presence of Ahr2, but not Cyp1a. Instead, Cyp1a would seem to serve a protective role, reducing the incidence of more severe morphological effects from exposure to 8-MBaP and preventing mortality.

These results agree with the findings for the BkF-induced x-fin, which was Ahr2 but not Ahr1a or Ahr1b dependent (Garland et al. 2020). Additionally, hepatic metabolism was not necessary for the development of the BkF-induced x-fin, determined by hepatic ablation (Garland et al. 2020). Although hepatic ablation does not equate to *cyp1a* KO, it does suggest that disruption to hepatic metabolism did not impact the formation of the BkF-induced x-fin. Another study by Fang et al. (2023) used co-exposure with an AHR antagonist (CH223191) to investigate the role of the AHR in the morphological effects of 8-MBaP. This study found that there was no impact from co-exposure on the morphological effects of 8-MBaP, whereas other PAHs did see reductions in toxicity due to co-exposure. However, the authors hypothesized that, due to the potency of 8-MBaP, a higher concentration of CH223191 than possible in their assay may have been necessary to see a reduction in morphological effects. Given the phenotypic similarity and Ahr2-dependence/cyp1a-independence of BkF and 8-MBaP, we hypothesize that x-fin-inducing PAHs act through the same mechanism.

The toxicity of AHR-dependent chemicals is often linked to the induction of cytochrome P450 enzymes, which catalyze the generation of reactive metabolites. Although harmful metabolites can form along the route to xenobiotic elimination, it evolved as an adaptive response. Our results showed Ahr2 dependence of 8-MBaP toxicity but independence from the canonical Cyp1a metabolism induced toxicity; i.e. 8-MBaP was more toxic in Cyp1a-KO larvae. Thus, some other Ahr2-dependent mechanism was operant. Ahr2 agonism is linked to impacts on a variety of genes in a ligand-specific manner, including estrogen and Wnt signaling, whose disruption during development can have detrimental impacts (Shankar et al. 2020). Both TCDD and retene are examples of other chemicals that cause Ahr2-dependent/Cyp1a-independent developmental effects in zebrafish, though neither causes the x-fin phenotype (Wilson et al. 2022; Rude et al. 2024). It is possible that either 8-MBaP or a Cyp1a-independent metabolite induces ligand-specific Ahr2-dependent transcriptomic changes leading to the x-fin phenotype.

### Phenotype emergence time course

**Fig. 3.**
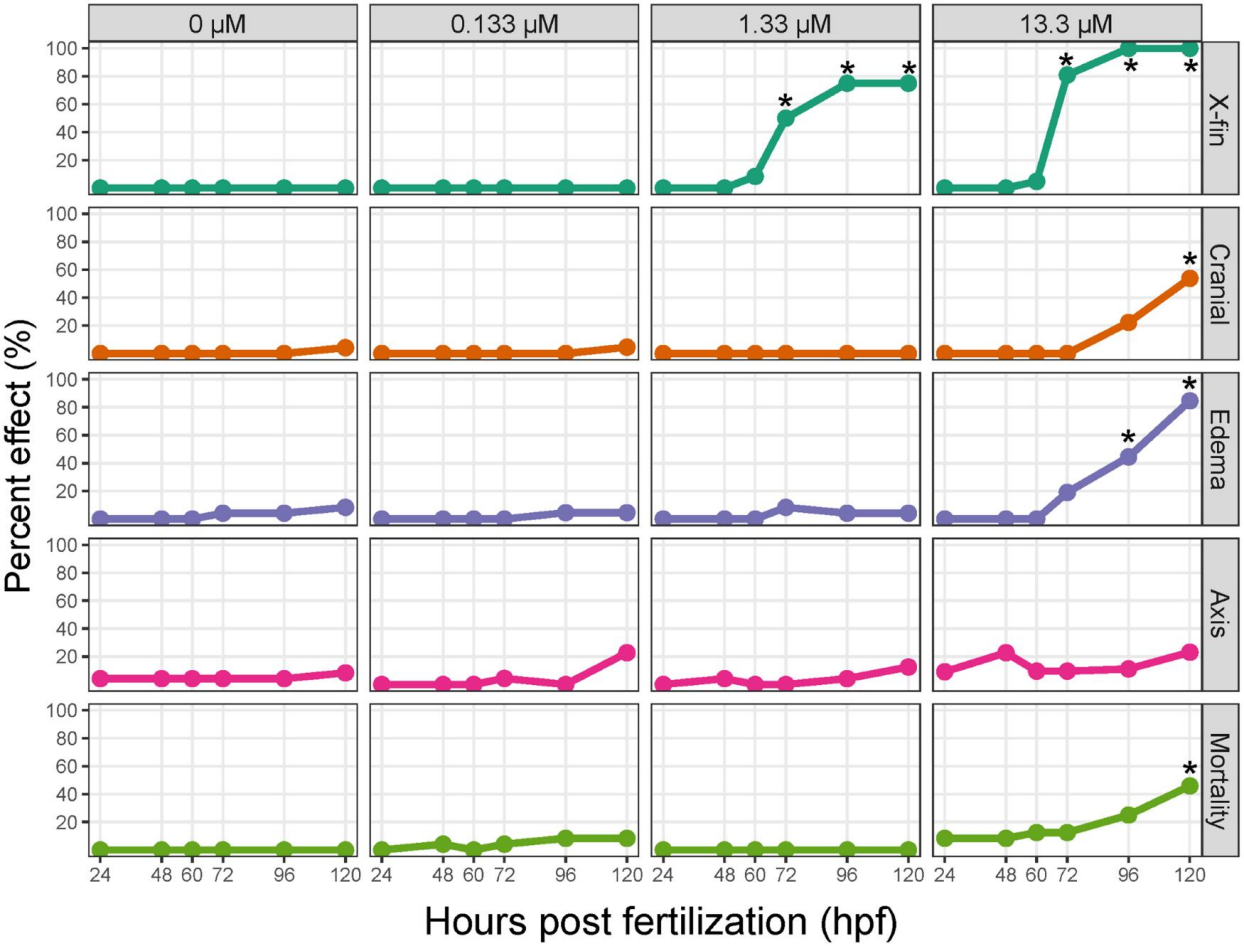
Time-course of morphological phenotype emergence following embryonic exposure to 8-methylbenzo[a]pyrene at 3 concentrations (0.133, 1.33 and 13.3 µM). Statistical significance determined using Fisher’s exact test with a Holm’s multiple comparison correction (asterisk indicates *P*-value ≤0.05).

The emergence of the x-fin phenotype was assessed over time in 8-MBaP-exposed larvae. Concentrations were chosen to span a range of predicted morphological effects at 120 hpf based on the concentration response curve in Fig. 1. The lack of morphological effects at 0.133 µM was as expected, and the 120 hpf morphological effects at 1.33 and 13.3 µM were as expected, aside from an increase in mortality above background levels and the lack of significant axis effects for 13.3 µM. However, the incidence of axis effects was higher than the control (Fig. 3).

**Fig. 4.**
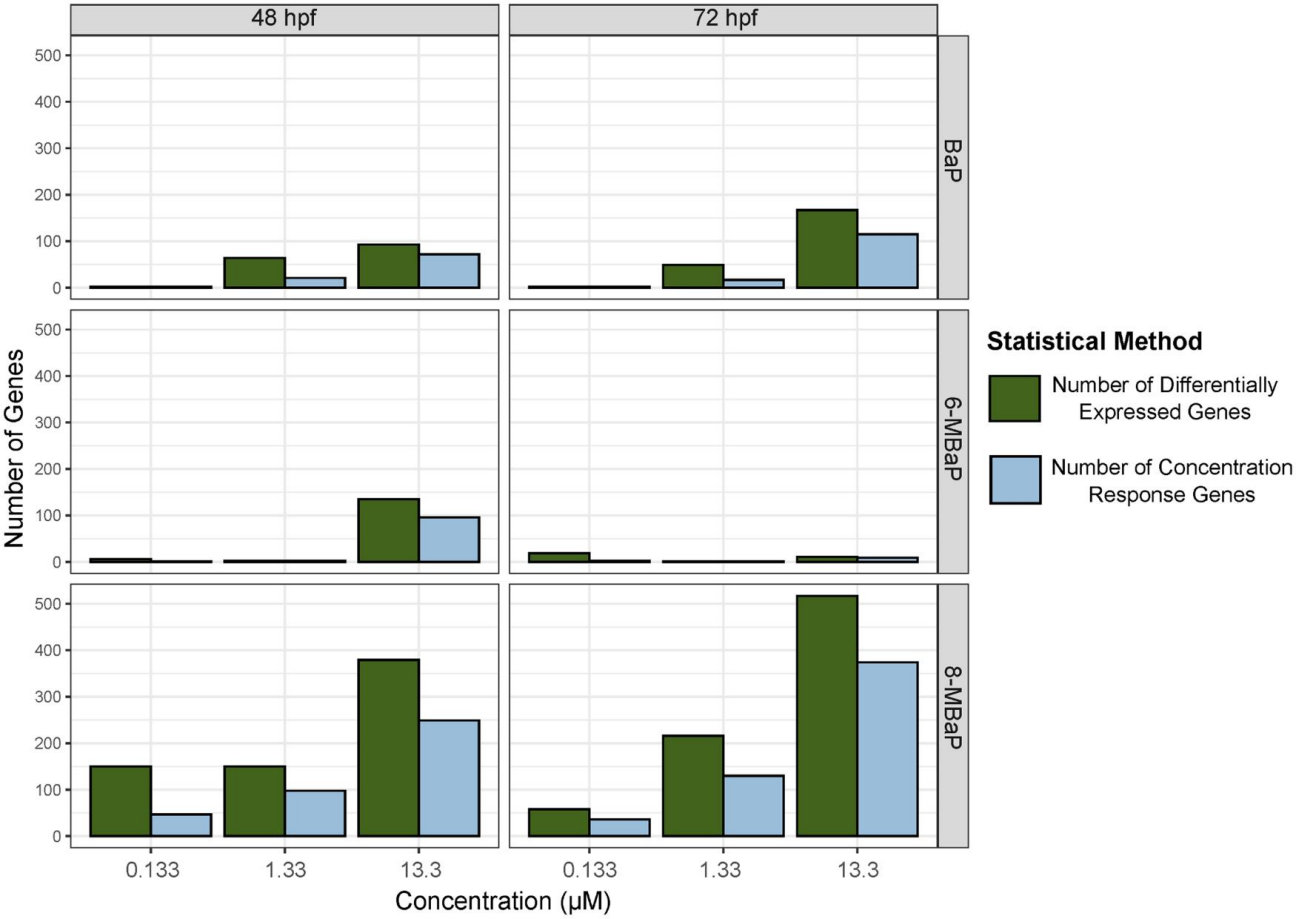
Bar plot showing the number of differentially expressed genes (adjusted *P*-value ≤0.05) and genes with a monotonic concentration response across concentration (0.133, 1.33, 13,3 µM) and timepoint (48 and 72 h post-fertilization [hpf]) for benzo[a]pyrene (BaP), 6-methylbenzo[a]pyrene (6-MBaP), and 8-methylbenzo[a]pyrene (8-MBaP). RNA extracted from pools of 6 whole embryos at 48 and 72 hpf, *n* = 4.

The x-fin was the earliest detectable morphological phenotype with effects above background, first observed at 72 hpf for both 1.33 µM (50%) and 13.3 µM (80%). The percent incidence increased to 75% and 100% by 96 hpf, respectively, and remained there at 120 hpf (Fig. 4). Edema was first detected above background for 13.3 µM at 96 hpf (44%), increasing to 85% by 120 hpf (Fig. 3). The cranial and mortality effects at 13.3 µM were 54% and 46%, respectively, at 120 hpf (Fig. 3). At 1.33 µM, no morphological effects were observed aside from x-fin, which was seen in 75% of larvae, 28% higher than expected based on concentration response modeling in Fig. 1A (Fig. 3).

The emergence of the BkF-induced x-fin phenotype described by Garland et al. (2020) was first detected at 60 hpf, 12 h earlier than the 8-MBaP-induced x-fin phenotype in the present study. Different timing was not unexpected for such different chemical structures. Toxic metabolites of 8-MBaP may take longer to form, or their rates of uptake may vastly differ.

### Transcriptomics

Transcriptomics were assessed in response to the 3 chemicals at 48 and 72 hpf and at 3 concentrations (0.133, 1.33, and 13.3 µM). Timepoints were chosen based on the findings in the “Phenotype emergence time course” section to ensure sampling before the emergence of the x-fin phenotype (48 hpf) and after x-fin emergence but before the emergence of other adverse morphological effects (72 hpf). The 3 concentrations were anchored to variations in morphological effects at 120 hpf: 0.133 µM (no effects), 1.33 µM (only x-fin), and 13.3 µM (x-fin and other morphological effects). Additional embryos were concurrently exposed and treated identically to plates used for transcriptomic sample collection to confirm that the expected incidences of 120 hpf phenotypes occurred. We observed the expected lack of morphological effects from BaP and 6-MBaP (Fig. S6). Morphological effects were as expected for each concentration of 8-MBaP, aside from a decrease in mortality at 13.3 µM compared with results in the “Phenotype emergence time course” section (Fig. S6).

#### 8-MBaP transcriptomic effects increased with concentration and time

8-MBaP had the highest number of DEGs at each concentration and timepoint. At 48 hpf, 0.133 µM 8-MBaP had more DEGs than BaP or 6-MBaP exposed to 2 orders of magnitude more chemical (13.3 µM) (Fig. 4). The majority of the 8-MBaP DEGs at 48 hpf for 1.33 µM and 13.3 µM (60% and 72% respectively) had a monotonic concentration response relationship, while only 31% were monotomic for 0.133 µM at 48 hpf (Fig. 4). The number of DEGs induced by 8-MBaP was higher at 72 hpf than 48 hpf for 1.33 µM and 13.3 µM (both concentrations that caused the x-fin phenotype by 72 hpf) in contrast to 0.133 µM for which there were fewer DEGs at 72 hpf than 48 hpf.

The 13.3 µM 6-MBaP exposure produced a similar temporal DEG trend to 8-MBaP at 0.133 µM, with a decrease in DEGs between 48 and 72 hpf. At 48 hpf, 6-MBaP (13.3 µM) was associated with 135 DEGs, but only 11 at 72 hpf. The lack of transcriptomic disruption by 6-MBaP compared with 8-MBaP further supported its inactivity and highlighted the impact of alkyl position on the biological activity of alkylated BaPs. BaP exposure resulted in a similar number of DEGs as 6-MBaP at 48 hpf. However, unlike 6-MBaP, the number of BaP-associated DEGs increased between 48 and 72 hpf at 13.3 µM. BaP (13.3 µM) was associated with 93 DEGs at 48 hpf, which increased to 167 by 72 hpf (Fig. 4). This indicated a lasting transcriptomic disruption by BaP throughout a crucial developmental window despite a lack of observed morphological outcomes.

#### Gene expression trends

**Fig. 5.**
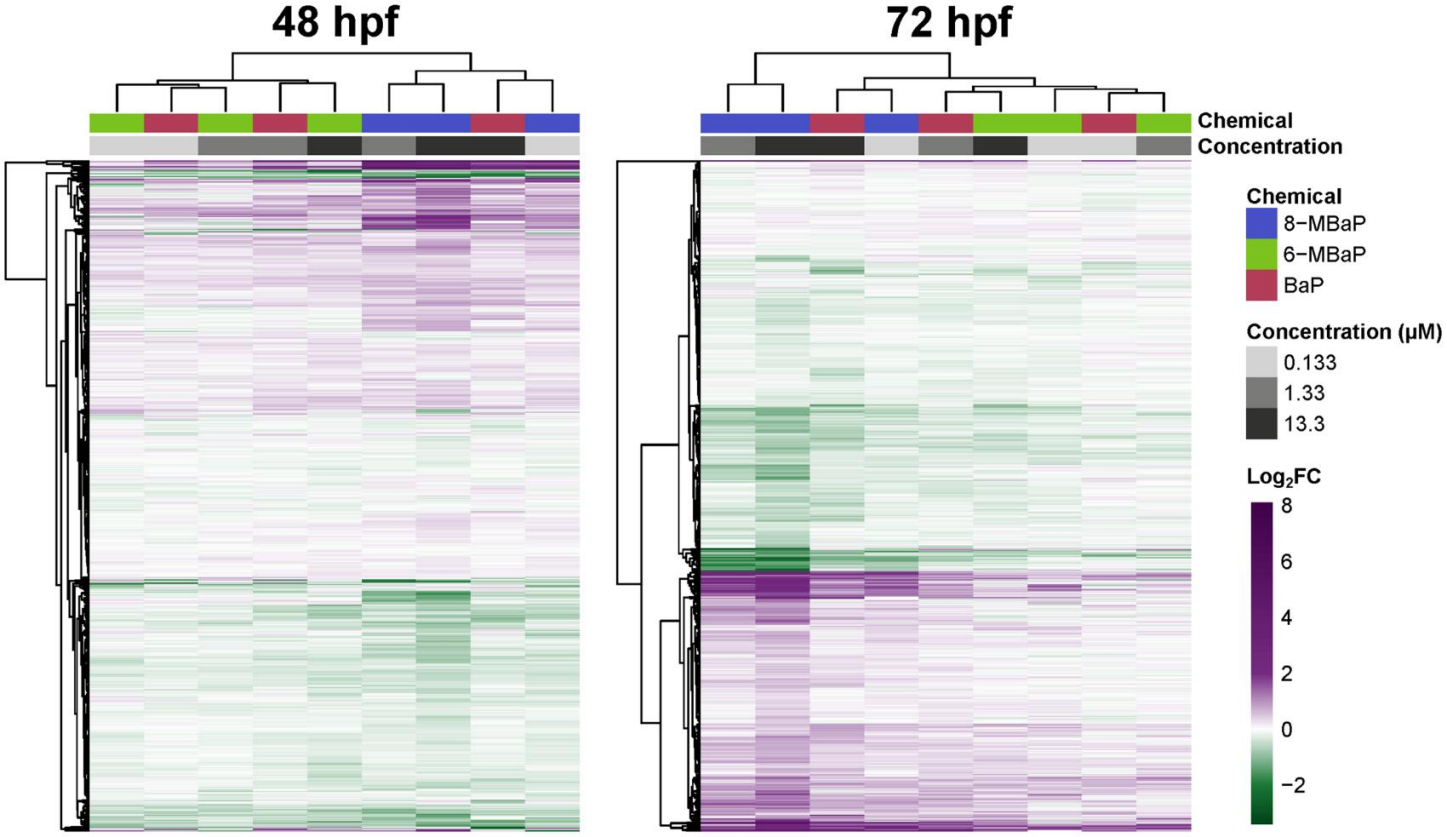
Clustered heatmap of the gene expression log_2_ fold change (log_2_FC) values for each gene that had a concentration response relationship for at least 1 chemical (8-methylbenzo[a]pyrene [8-MBaP], 6-methylbenzo[a]pyrene [6-MBaP], or benzo[a]pyrene [BaP]) at each timepoint (48 and 72 h post-fertilization [hpf]). RNA extracted from pools of 6 whole embryos at 48 and 72 hpf, *n* = 4.

Gene expression changes across chemicals and concentrations had similar directionality for the most part, despite differences in the number of DEGs, the number of monotonic concentration–response genes, and morphological outcomes (Fig. 5). At 48 hpf, 2 distinct clusters emerged: One for 6-MBaP exposures and lower concentrations of BaP with smaller log_2_FC values and another for 8-MBaP exposures and the highest concentration of BaP with larger log_2_FC values. The cluster with larger log_2_FC values was further divided into treatments with morphological effects (8-MBaP 1.33 µM and 13.3 µM) and those without (8-MBaP 0.133 µM and BaP 13.3 µM). By 72 hpf, this clustering changed, as the 2 higher concentrations of 8-MBaP that caused x-fin became more distinct, clustering separately from the other treatments.

#### X-fin-associated genes

X-fin-associated genes were identified with a BMC below 1.33 µM in 8-MBaP exposure; ∼80% of larvae exposed to 1.33 µM 8-MBaP displayed the x-fin phenotype at 120 hpf, so genes with higher BMCs were unlikely to be involved in the x-fin phenotype. Instead, these genes were more likely related to phenotypes specific to higher concentrations (Edema, Axis, and Cranial). We performed functional enrichment analysis of x-fin-associated genes at 48 and 72 hpf to understand how the roles of disrupted genes shifted before and after the appearance of the x-fin.

**Fig. 6.**
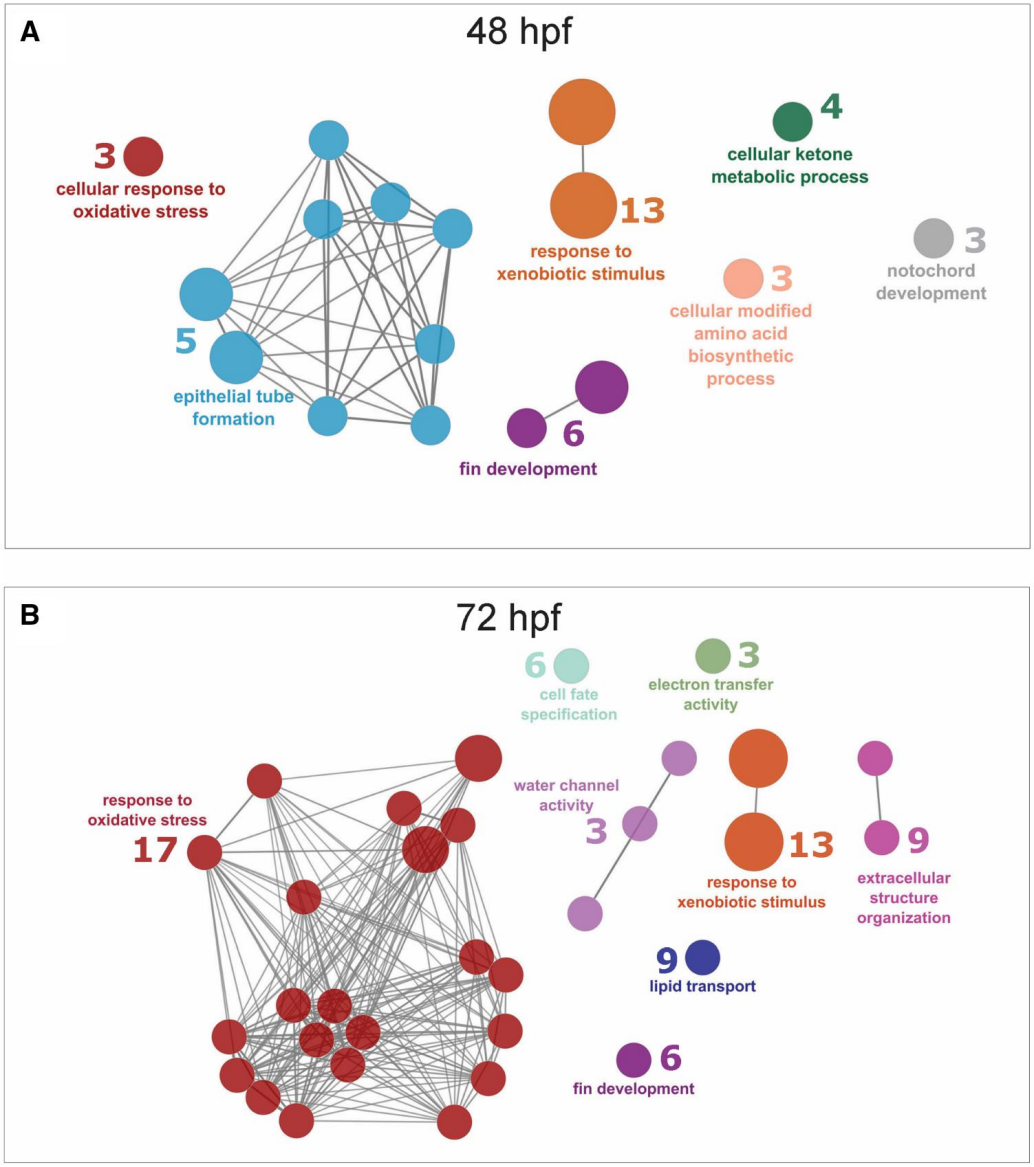
Results from functional enrichment analysis using GlueGo for x-fin-associated genes at (A) 48 h post-fertilization (hpf) and (B) 72 hpf. The x-fin-associated gene set includes genes with benchmark concentrations (BMCs) ≤1.33 µM for 8-methylbenzo[a]pyrene (8-MBaP). Nodes represent Gene Ontology Biological Process terms enriched in the gene set (*P*-value ≤ 0.05); nodes are connected based on >50% overlap in genes. Functional groups are labeled with the number of genes represented by the cluster and the GO: BP term with the lowest enrichment *P*-value. RNA extracted from pools of 6 whole embryos at 48 and 72 hpf, *n* = 4.

Several functional groups were uniquely enriched at 48 hpf, including cellular ketone process, epithelial tube formation, notochord development, and cellular modified amino acid biosynthetic process (Fig. 6A). The cellular ketone metabolic process functional group may be associated with the oxidative stress functional group, as ketones can play an integral role in the response to oxidative stress (Rojas-Morales et al. 2020). *Anxa1c*, e.g. is indicated as a member of both the cellular oxidative stress response and the cellular ketone metabolism process. The epithelial tube formation group included 3 genes specifically related to otic vesicle development (*dlx3b*, *fsta*, *tfap2c*) with increased expression. These genes have no described involvement in fin development. The notochord development group included *ccn2a*, *ccn2b*, and *igf2b*, all of which had increased expression. KO of *ccn2a* causes bent tail, curved body, and cardiac edema phenotypes (Singh et al. 2025)*. Ccn2a* expression is increased in zebrafish heart regeneration, as it promotes extracellular matrix-stabilizing genes (Mukherjee et al. 2021).

Functional groups unique to 72 hpf include water channel activity, electron transfer activity, extracellular structure organization, lipid transport, and cell specification (Fig. 6B). The water channel activity functional group includes *mipa and mipb*, water transmembrane transporters important for the development of the eye and lens transparency (He et al. 2023). Most genes (5/9) in the extracellular organization functional group were collagen type 1 and 2 genes important for fin morphogenesis, all of which had decreased expression following 8-MBaP exposure (Bretaud et al. 2019). *And1* and *and2*, both genes included in the fin development functional group, which had decreased expression at 48 hpf, are essential for the formation of actinotrichia, collagen structures present in the fins. These genes may have upstream involvement in the reduction in collagen genes at 72 hpf. The cell fate specification functional group included 6 genes, including *dlx3b* and *tfap2c*, genes involved in otic vesicle development, which were also differentially expressed in the same direction at 48 hpf, suggesting persistent disruption of otic vesicle development. Embryonic and larval behavioral assessments could identify phenotypic manifestations of this disruption. The electron transfer activity and lipid transport functional groups are closely associated with a response to oxidative stress, with the latter showing the greatest number of genes at 72 hpf. Electron transfer is often implicated in the creation of reactive oxygen species and oxidative stress (Kovacic and Jacintho 2001). Of the 3 genes in this functional group, *gsr* was also included in the oxidative stress response functional group. Similarly, the disruption of lipid homeostasis and lipid membranes is often a downstream impact of an overwhelmed oxidative stress response (Laguerre et al. 2017). *Abxa1c*, a key gene involved in oxidative stress response, is also a member of the lipid transport functional group.

Overlap between 48 and 72 hpf enriched functional groups included response to oxidative stress, response to xenobiotic stimulus, and fin development. There was substantial overlap between genes in the response to xenobiotic stimulus (11/13) and fin development (3/6) functional groups across timepoints. Shared genes in the fin development functional group included *Ahr2*, *bhlha9*, and *sp9*. Only 1 gene was shared between 48 and 72 hpf in the functional group for the response to oxidative stress (*abxa1c*). Some of the genes related to oxidative stress at 72 hpf also had a concentration response to BaP exposure (7/17) at 72 hpf, but none responded to 6-MBaP. For genes exhibiting a concentration response shared between 8-MBaP and BaP, the log2FC for 8-MBaP was larger in most of the cases (Fig. S7). Oxidative stress and DNA damage caused by reactive metabolites formed by AHR-induced CYP enzymes is the canonical pathway of BaP toxicity and was previously proposed to explain the morphological activity of 8-MBaP, which did not include the x-fin phenotype (Elfawy et al. 2021; Fang et al. 2023). The observed oxidative stress response is likely caused by buildup of oxidative stress due to reactive metabolite formation and subsequent overwhelm of antioxidative capacity.

Although 8-MBaP elicited a stronger oxidative stress response than either BaP or 6-MBaP, reactive metabolite-induced oxidative stress was not unique to 8-MBaP. It was thus unlikely to play a causal role in the development of the x-fin. Persistent oxidative stress affects normal developmental processes and may lead to the morphological phenotypes observed at higher concentrations (cranial malformations, edema, and axis), previously observed in BaP exposure as well (Parlak 2018; Elfawy et al. 2021; Zhang et al. 2025). Although oxidative stress–response genes had BMCs below 1.33 µM and were included in the x-fin-associated genes, the BMC was the concentration at which expression of these genes was 1.35 SDs from control expression. Meeting this threshold did not necessarily mean that the gene was linked to morphological outcomes at that concentration.

#### X-fin unique genes

48 hpf x-fin-associated genes were further filtered to x-fin unique, excluding genes that also had a concentration–response relationship to either BaP or 6-MBaP. This allowed us to exclude any genes unrelated to the x-fin that might have been generalized to the xenobiotic response or to other transcriptomic effects of chemicals with similar structures, such as reactive metabolite-induced oxidative stress. The window of susceptibility for BkF-induced x-fin formation is 12 to 36 hpf, indicating that the molecular initiating event occurs before 36 hpf, followed by phenotypic emergence by 60 hpf or, in the case of 8-MBaP, 72 hpf (Garland et al. 2020). To investigate gene expression changes after the molecular initiating event and before the emergence of the phenotype, we focused on the 48 hpf timepoint.

**Fig. 7.**
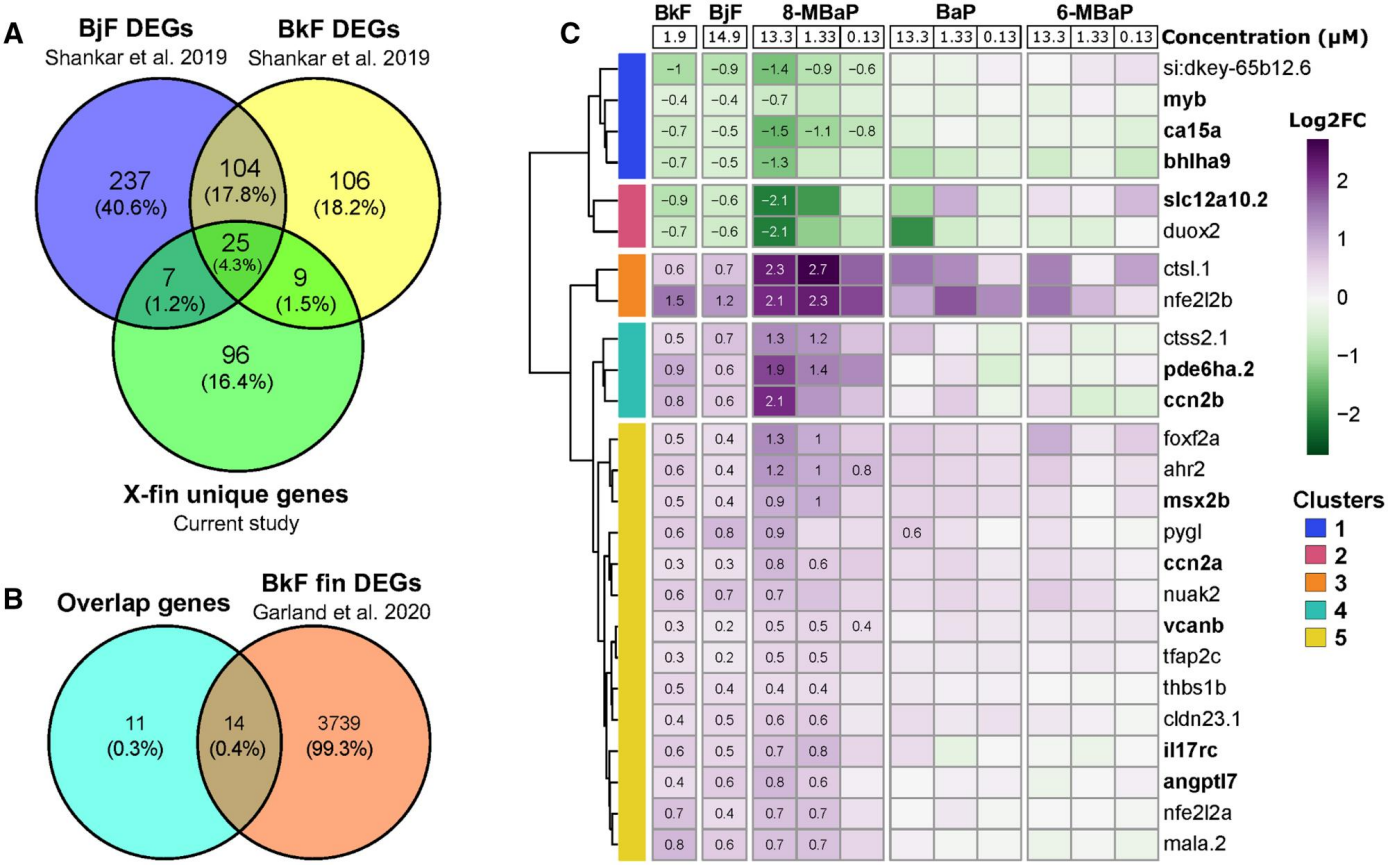
A) Venn diagram comparing x-fin unique genes from the current study (RNA extracted from pools of 6 whole embryos at 48 hpf, *n* = 4) against differentially expressed genes (DEGs) from Shankar et al. 2019 for benzo[*j*]fluoranthene (BjF) and benzo[*k*]fluoranthene (BkF) exposures (RNA extracted from pools of 8 whole embryos at 48 hpf, *n* = 4). B) Venn diagram comparing genes overlapping between all groups in (A) (*n* = 25/4.7%) with fin tissue specific DEGs for BkF exposure from Garland et al. 2020 (RNA extracted from pools of 50 embryo caudal fins at 48 hpf, *n* = 4). C) Heatmap of the log_2_FC for genes overlapping between all groups in (A) (*n* = 25/4.7%) for BjF and BkF and all treatments in the current study. Bolded gene names indicate genes unique to bulk sequencing determined in (B). Clusters were determined by hierarchical clustering.

X-fin unique genes were compared with DEGs identified at 48 hpf in BkF- and BjF-exposed embryos by Shankar et al. (2019) (Fig. 7A). BjF caused the same x-fin phenotype observed from BkF and 8-MBaP exposures. Overlap among 8-MBaP, BjF, and BkF would help identify genes specifically associated with the x-fin phenotype. Twenty-five genes were shared between all treatments (Fig. 7A). Recurrent differential expression of these genes across chemicals and studies provided high confidence in their involvement in the x-fin phenotype and supported our hypothesis that these chemicals act through the same mechanism. This set of 25 genes was compared against the caudal fin-specific DEGs at 48 hpf from BkF exposure, identified by Garland et al. (2020) (Fig. 7B). Caudal fin-specific DEGs were identified by RNA sequencing of caudal fin tissue of control fish and BkF-exposed fish with the x-fin phenotype. Sequencing the malformed caudal fin tissue resulted in a higher number of DEGs than whole-embryo sequencing. Despite the greater number of caudal fin-specific DEGs, 11 genes were differentially expressed in whole-embryo sequencing but not in fin samples. These genes may have an upstream role in the emergence of the x-fin phenotype but not in the caudal fin tissue itself (Fig. 7B). These genes are bolded in the heatmap comparing the log_2_FC for each of these overlap genes across chemicals (Fig. 7C). Clusters 1, 2, and 4 had the most distinct gene expression trends for x-fin-specific genes compared with BaP and 6-MBap (Fig. 7C).

##### Cluster 1

This cluster included 4 genes with reduced expression associated with all x-fin-causing chemicals and minimal insignificant log_2_FCs for 6-MBaP and BaP. Three of these genes were not differentially expressed in fin tissue (*myb*, *ca15a*, and *bhlha9*). *Si: dkey-65b12.6* is an uncharacterized gene whose mRNA and protein expression are increased in the proximal region of the adult caudal fin (Rabinowitz et al. 2017). Despite not being differentially expressed by BaP and 6-MBaP exposures, *si: dkey-65b12.6* was amongst the top 5 most reduced transcripts for 4 other PAHs and thus not unique to x-fin-causing chemicals (Shankar et al. 2019). *Myb* is a highly conserved and well-characterized transcription factor playing an essential role in definitive hematopoiesis and B-cell differentiation (Soza-Ried et al. 2010). *Myb* is also characterized as a proto-oncogene whose expression is affected by many environmental contaminants, including benzene (Yu et al. 2011). *Ca15a* is a carbonic anhydrase involved in Na+ uptake with impacts on ion homeostasis and acid–base regulation (Lin et al. 2008). Embryonic treatment with sulfonamides, a class of chemicals with strong carbonic anhydrase inhibitor activity, causes otic vesicle malformations and pectoral and tail fin malformations due to increased apoptosis (Matsumoto et al. 2017). X-fin-associated genes had functional enrichment of otic vesicle genes at 48 hpf and continued disruption of some of these genes at 72 hpf, including *tfap2c* in cluster 5 (Fig. 7C). Prolactin and other hormones are key regulators of *ca15a* and ion homeostasis in zebrafish (Guh and Hwang 2017). BHLHA9 is a transcription factor that regulates ectodermal ridge formation in humans (Kataoka et al. 2018). Increased copy number is associated with congenital split hand/foot syndrome and long bone deficiency, whereas loss-of-function mutations are associated with syndactyly, characterized by webbed fingers in mammals (Schatz et al. 2014; Kataoka et al. 2018). In zebrafish, morpholino knockdown causes shortened pectoral fins and caudal fin malformations (Klopocki et al. 2012).

##### Cluster 2

This cluster included 2 genes with decreased expression (log_2_FC<−2) at 13.3 µM and variable directional responses in the BaP- and 6-MBaP-exposed embryos. *Slc12a10.2*, a solute carrier key in ion uptake of Na+ and Cl− in the gills, with regulation of its expression linked closely to *ca15a*, was not differentially expressed in fin tissue (Wang et al. 2009). Like *ca15a*, *slc12a10.2* is regulated by a variety of hormones, including prolactin (Shu et al. 2018). KO of *slc12a10.2* leads to increased *ca15a* and Na+ content in embryonic zebrafish, followed by larval lethality in freshwater due to lack of Cl− uptake (Chang et al. 2013; Shu et al. 2018). Instead of a compensatory increase in *ca15a* expression, both *slc12a10.2* and *ca15a* had decreased expression in all x-fin-inducing chemical exposures, indicating a disruption to tightly regulated ion homeostasis. *Slc12a10.2* was amongst the top 10 genes with reduced expression in embryos exposed to BkF and BjF, and not in the other 4 PAHs tested in the same study (Shankar et al. 2019). *Duox2* is an NADPH oxidase, a primary source for ROS production necessary in both thyroid hormone synthesis and wound healing. KO of Duox2 results in zebrafish exhibiting hypothyroidism, ragged and split fins in adults, and delayed fin regeneration (Chopra et al. 2019, 2023; Sun et al. 2021). Fin morphology in larvae was not specifically addressed in KO or morpholino knockdown studies, so the phenotypic similarity to the x-fin observed in this study is unknown. The assessment of thyroid hormone concentrations in 8-MBaP-exposed embryos/larvae would help confirm the biological consequences of decreased *duox2* expression.

##### Cluster 4

This cluster included 3 genes with increased expression associated with x-fin-inducing chemicals and variable directional responses from BaP and 6-MBaP. Two of these genes were not differentially expressed in fin tissue (*pde6ha.2* and *ccn2b*). *Ctss2.1* is a lysosomal cysteine-type protease important in immune response and often used as an allergic inflammatory biomarker (Vaz-Rodrigues et al. 2025). Its increased expression suggested an immune response unique to exposure to x-fin-inducing chemicals. *Pde6ha.2* encodes for an inhibitory subunit of the phosphodiesterase 6 (pde6) protein, which has circadian oscillations that suggest its involvement in regulating photopic sensitivity (Abalo et al. 2020). Increased expression of *pde6ha.2* is seen during the day, along with increased photosensitivity (Abalo et al. 2020). *Ccn2b* is an essential gene in notochord development, along with *ccn2a*, included in cluster 5, also with increased expression. *Ccn2* genes are involved in the response to cardiac injury and intervertebral disk homeostasis (Mukherjee et al. 2021; Rayrikar et al. 2023). *Ccn2a* specifically is associated with increased collagen gene expression at the site of cardiac injury; however, collagen type 1 genes had decreased expression at 72 hpf (Mukherjee et al. 2021).

## Conclusions

We demonstrated distinct developmental toxicity for 8-MBaP compared with the parent BaP and another alkylated derivative, 6-MBaP. Although neither 6-MBaP nor BaP produced morphological effects at the tested concentrations, 8-MBaP caused a potent caudal fin malformation (x-fin) at low concentrations and edema, cranial and axial malformations at higher concentrations. These effects were Ahr2 dependent but Cyp1a independent.

Transcriptomic analyses further distinguished 8-MBaP from 6-MBaP, revealing minimal effects of 6-MBaP exposure and a concentration- and time-dependent transcriptomic disruption from 8-MBaP. Although BaP did not induce morphological effects, we found evidence of persistent transcriptomic disruption by BaP during a crucial developmental window.

Functional enrichment analysis of genes disrupted by 8-MBaP revealed a minor oxidative stress response at 48 hpf, followed by a larger response at 72 hpf. This finding aligns with a study published during the preparation of this manuscript, which proposes that oxidative damage from a minor metabolite of 8-MBaP, 3-OH-8-MBaP, underlies the compound’s potent developmental toxicity in zebrafish (Wang et al. 2025). However, the molecular initiating event for x-fin formation occurs prior to 36 hpf, whereas the observed increase in oxidative stress response occurs between 48 and 72 hpf. Thus, although metabolite-induced oxidative damage may contribute to the phenotypes at higher concentrations addressed by Wang et al. (2025), it is unlikely to explain the x-fin phenotype.

In addition to oxidative stress pathways, enrichment analysis revealed disruptions in otic vesicle development at 48 hpf, eye development at 72 hpf, and fin development at both timepoints. Examination of genes uniquely altered by x-fin-inducing chemicals highlighted disruptions in ion homeostasis, thyroid hormone synthesis, ROS production, and ectodermal ridge formation, all processes essential for normal fin development. Spatial transcriptomics paired with single-cell sequencing are ongoing to identify the cell type and location-specific contributions to the x-fin-specific transcriptomic changes identified in the current study. Ahr2’s role in regulating these x-fin-specific genes remains unclear. Future research should examine the causal relationship between the transcriptomic signature of x-fin-inducing chemicals and the established Ahr2 dependence of the phenotype.

## Supplementary Material

kfaf164_Supplementary_Data

## Data Availability

The targeted transcriptomic data discussed in this publication have been deposited in NCBI's Gene Expression Omnibus (Edgar et al. 2002) and are accessible through GEO Series accession number GSE308584. Additional data is available in supplemental materials.
