# Supplementary material for: Position-specific methyl substitution on benzo[a]pyrene drives AHR-dependent fin duplication in zebrafish
Source: Toxicol Sci. 2025 Nov 20;209(1):kfaf164. doi: 10.1093/toxsci/kfaf164 (PMC12826582; doi:10.1093/toxsci/kfaf164)
Supplement: kfaf164_Supplementary_Data [file kfaf164_supplementary_data.zip › Supplemental Figures.docx]

Position-Specific Methylation of Benzo[a]pyrene Drives AHR-Dependent Fin Duplication in Zebrafish

Authors: Mackenzie L. Morshead, Lisa Truong, Robyn L. Tanguay


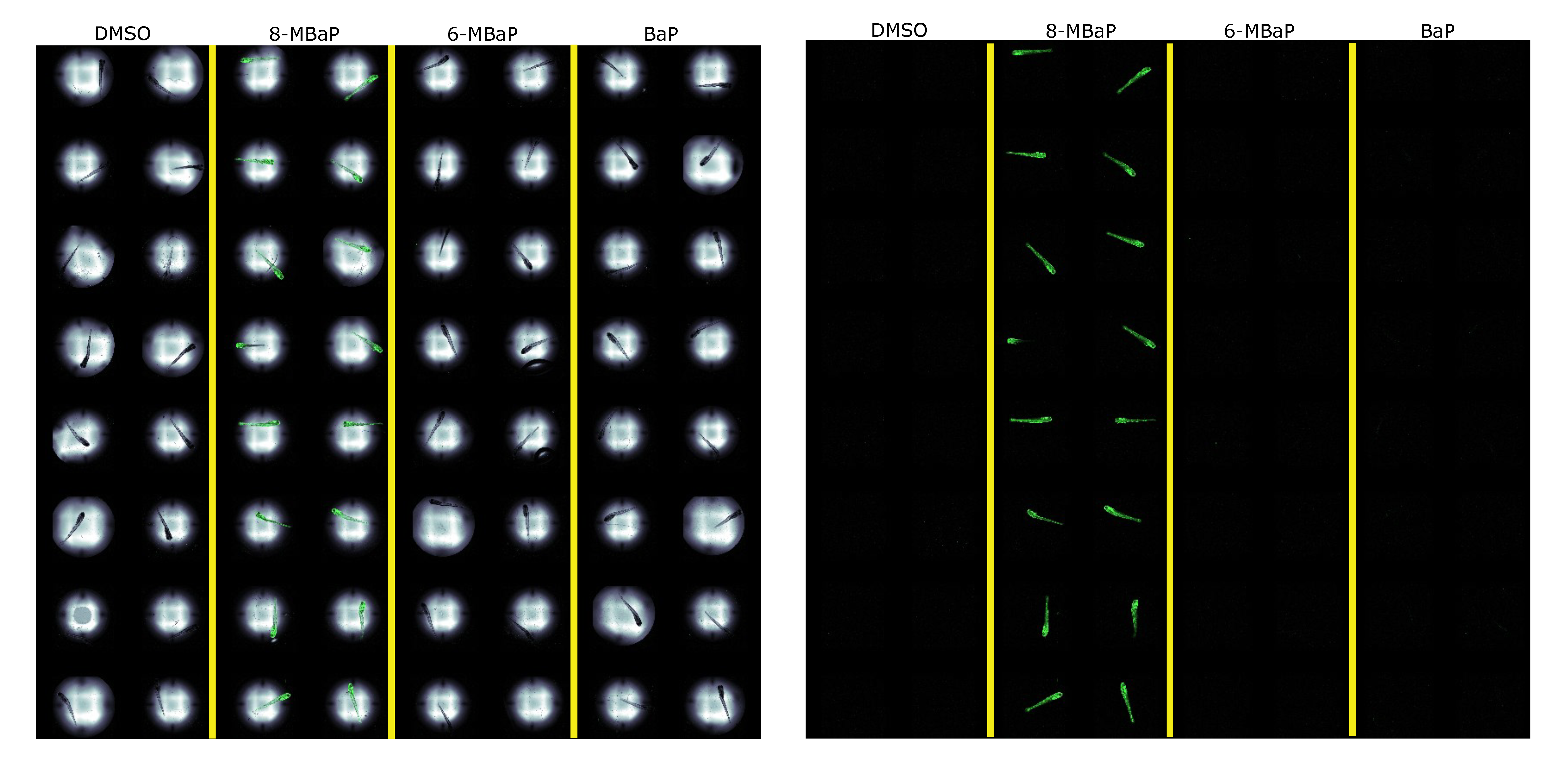


**Supplemental figure 2:** Images of *cyp1a* reporter fish at 120 hpf in a 96-well plate at 2X following embryonic exposure to DMSO, 8-methybenzo(a)pyrene (8-MBaP), 6-methybenzo(a)pyrene (6-MBaP), or benzo(a)pyrene (BaP) at 1.33 µM (n=16). Brightfield images are overlayed GFP fluorescence on the left.


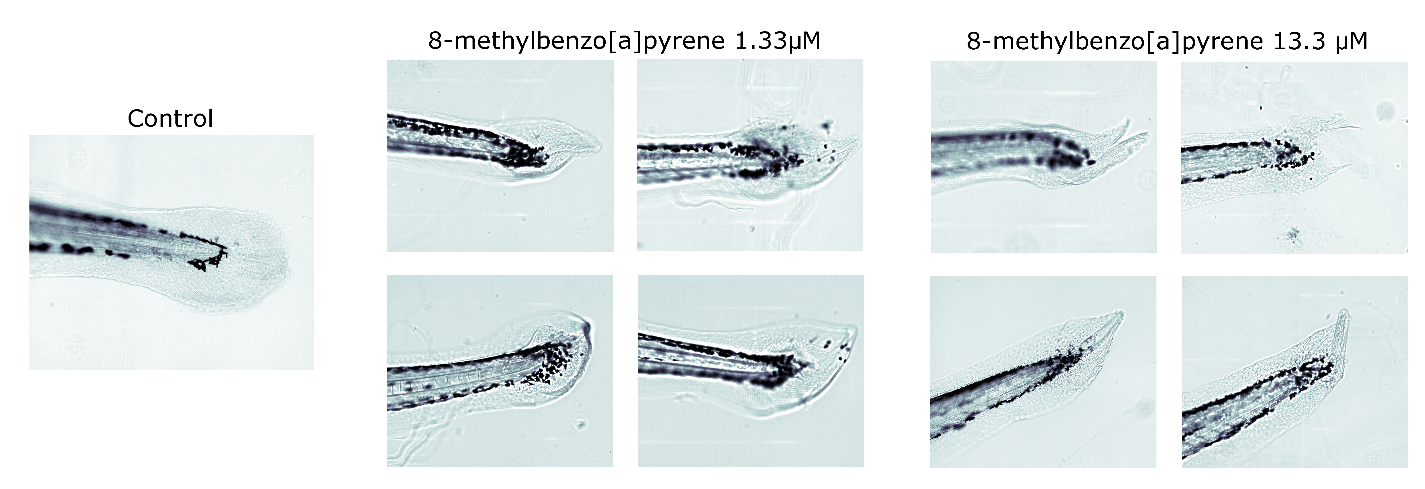


**Supplemental figure 1:** Representative images of the caudal fin malformation (x-fin) at 120 hpf following exposure to 8-methybenzo(a)pyrene at 1.33 µM and 13.3 µM.


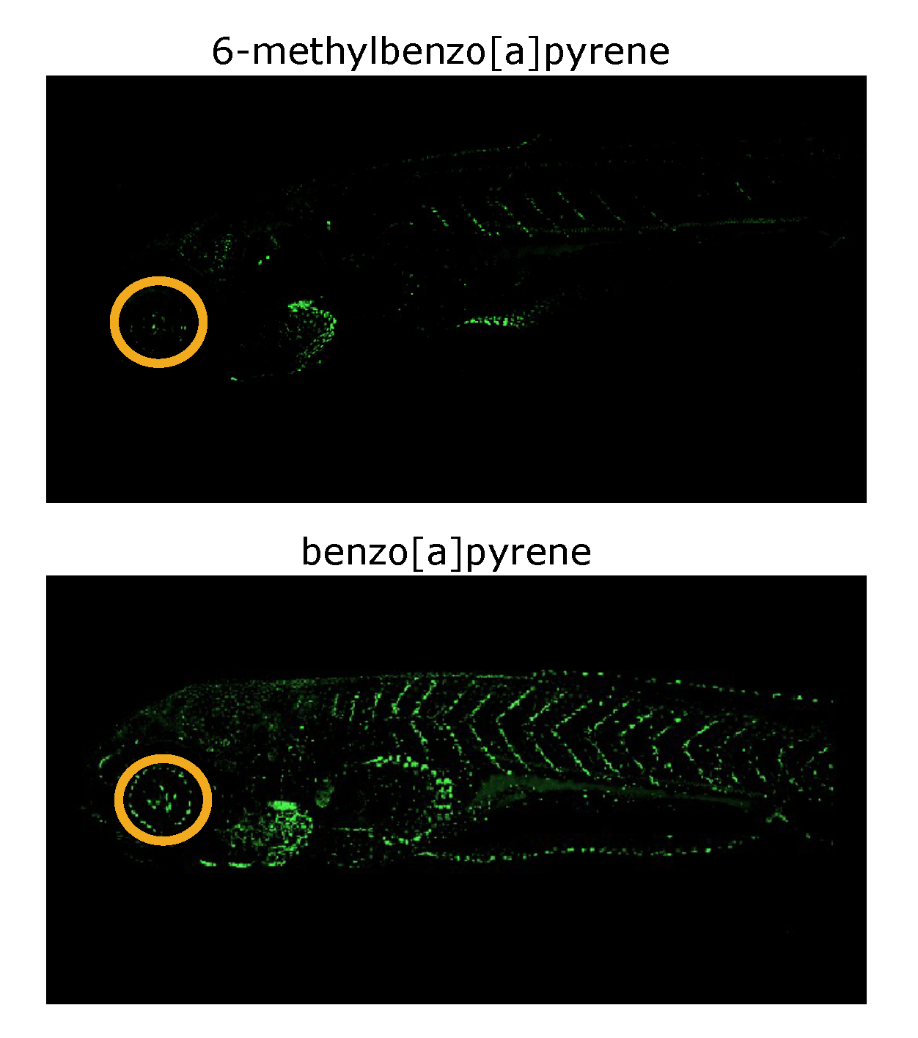


**Supplemental figure 3:** Representative images of *cyp1a* reporter fish at 120 hpf at 10X following embryonic exposure to 6-methybenzo(a)pyrene (6-MBaP) or benzo(a)pyrene (BaP) at 1.33 µM. Orange ring indicates the location of the eye.


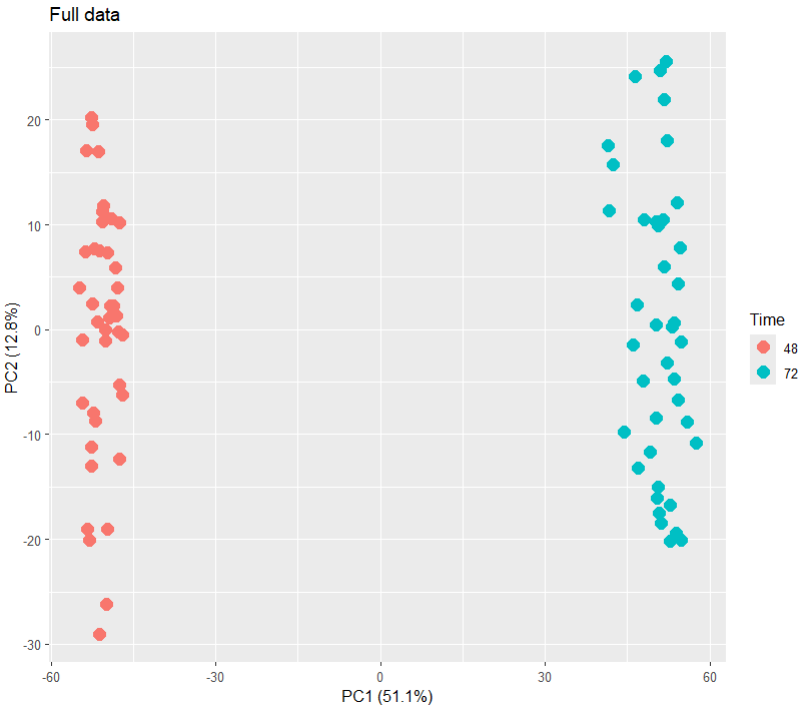


**Supplemental Figure 4:** Principal component analysis of log_2_ counts per million reads for all RNA samples collected at 48 hours post fertilization (hpf) and 72 hpf regardless of chemical treatment. RNA extracted from pools of 6 whole embryos at 48 and 72 hpf.


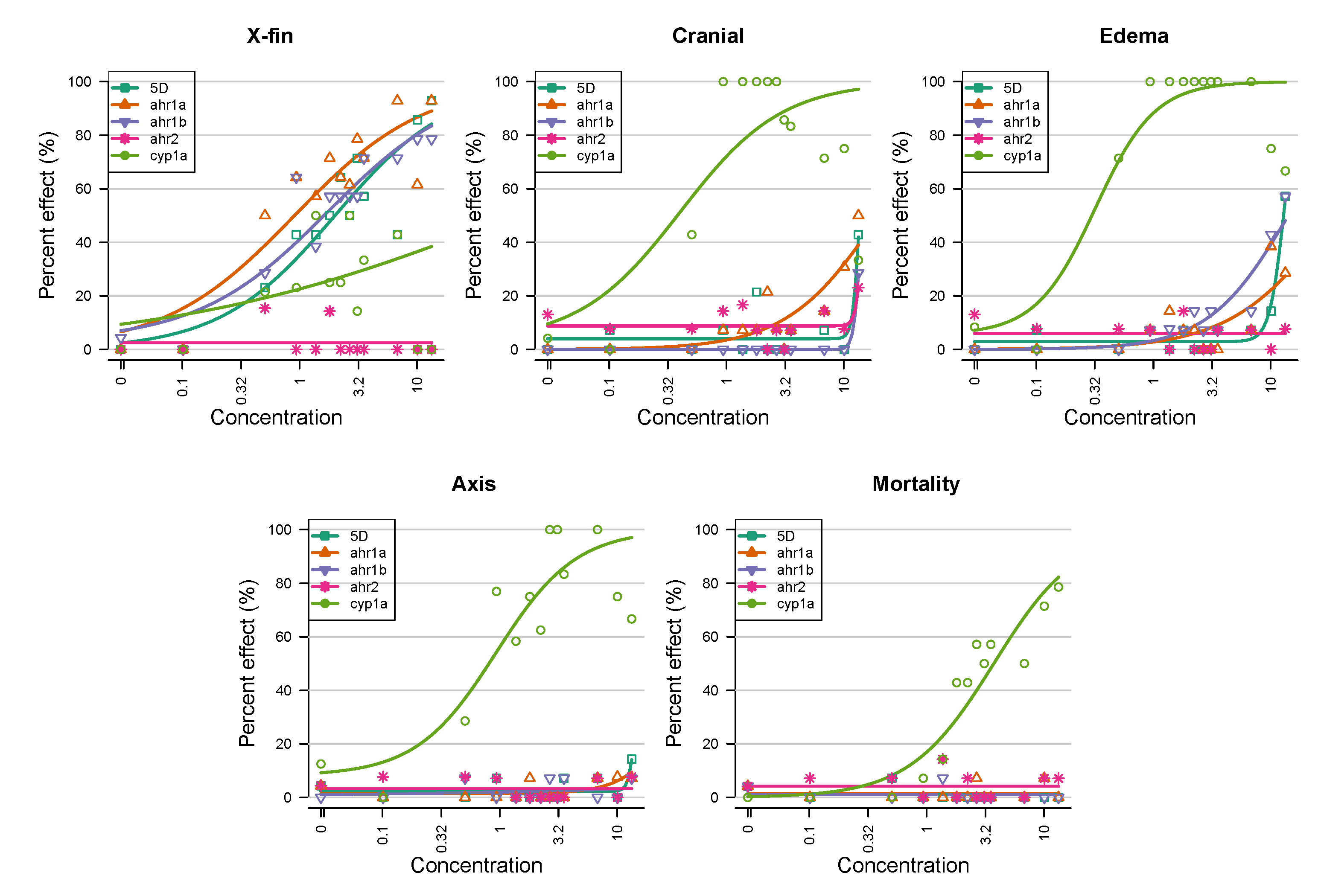


**Supplemental figure 5:** Concentration response curves for each effected morphological endpoint at 120 hours post fertilization (hpf) following embryonic exposure to 8-methylbenzo[a]pyrene from 7-120 hpf in wildtype (5D) and knockout lines for *ahr1a* (*ahr1a^osu6^*), *ahr1b* (*ahr1b^wh36^*), *ahr2* (*ahr2^hu3335^*), and *cyp1a* (*cyp1a^osu4^*).


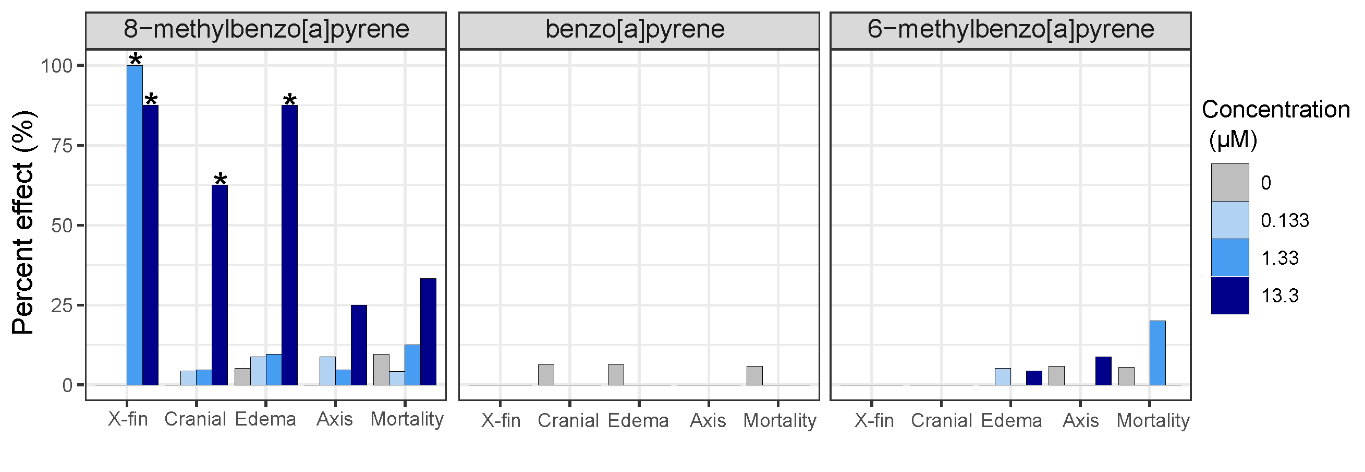


**Supplemental Figure 6:** Bar plot of the percent effect for each endpoint at 120 hours post fertilization (hpf) across concentration (0.133, 1.33, and 13.3 µM) for 8-methybenzo(a)pyrene, 6-methybenzo(a)pyrene, or benzo(a)pyrene in holdback plates co-currently exposed during exposures used for RNA collections to confirm expected morphological effects.


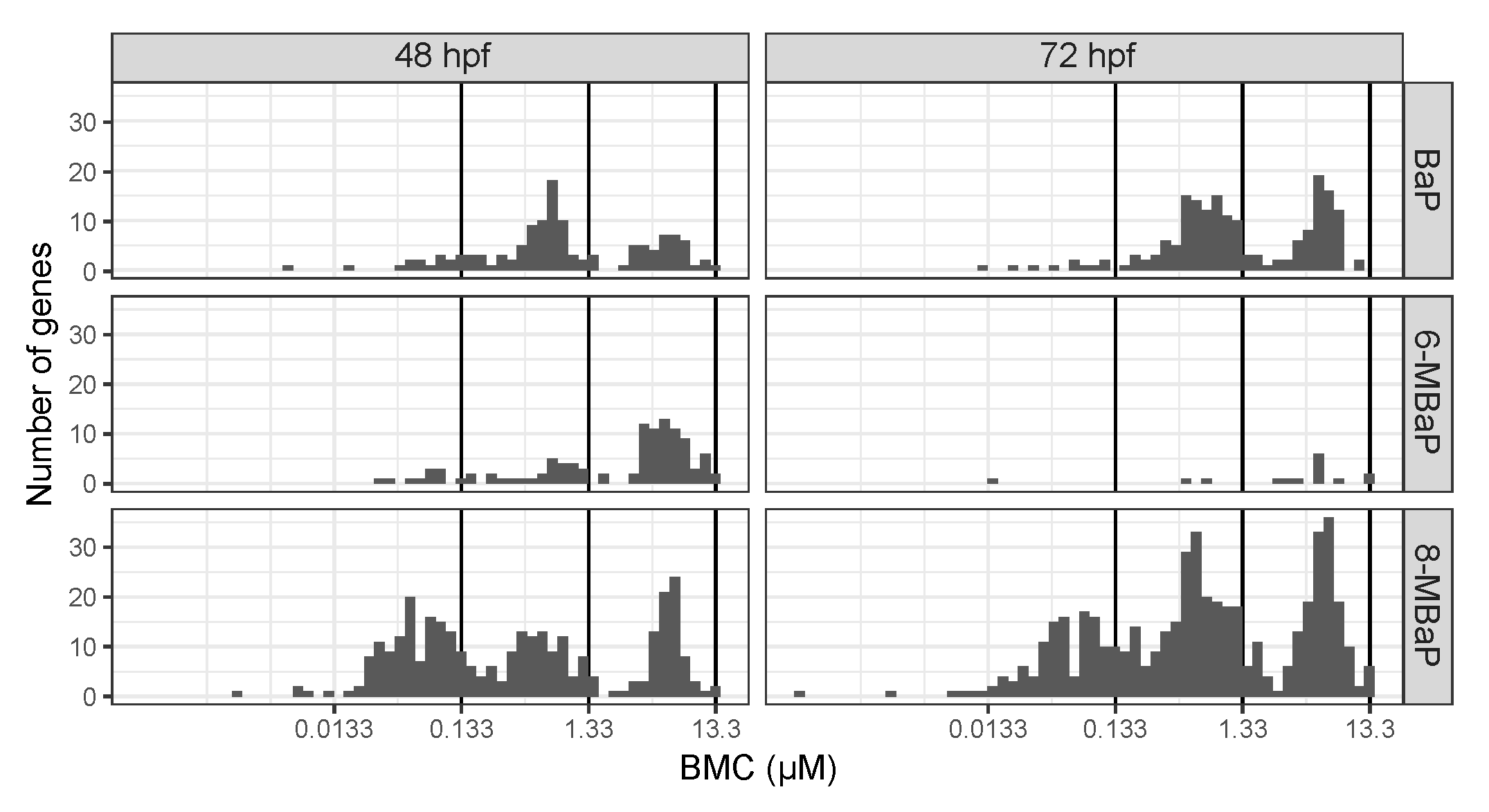
**Supplemental Figure 7:** Histograms of the number of genes at each benchmark concentration (BMC) at each time point, 48 hours post fertilization (hpf) and 72 hpf for each chemical 8-methybenzo(a)pyrene (8-MBaP), 6-methybenzo(a)pyrene (6-MBaP), or benzo(a)pyrene (BaP). Vertical lines indicate exposure concentrations used for RNA sequencing.


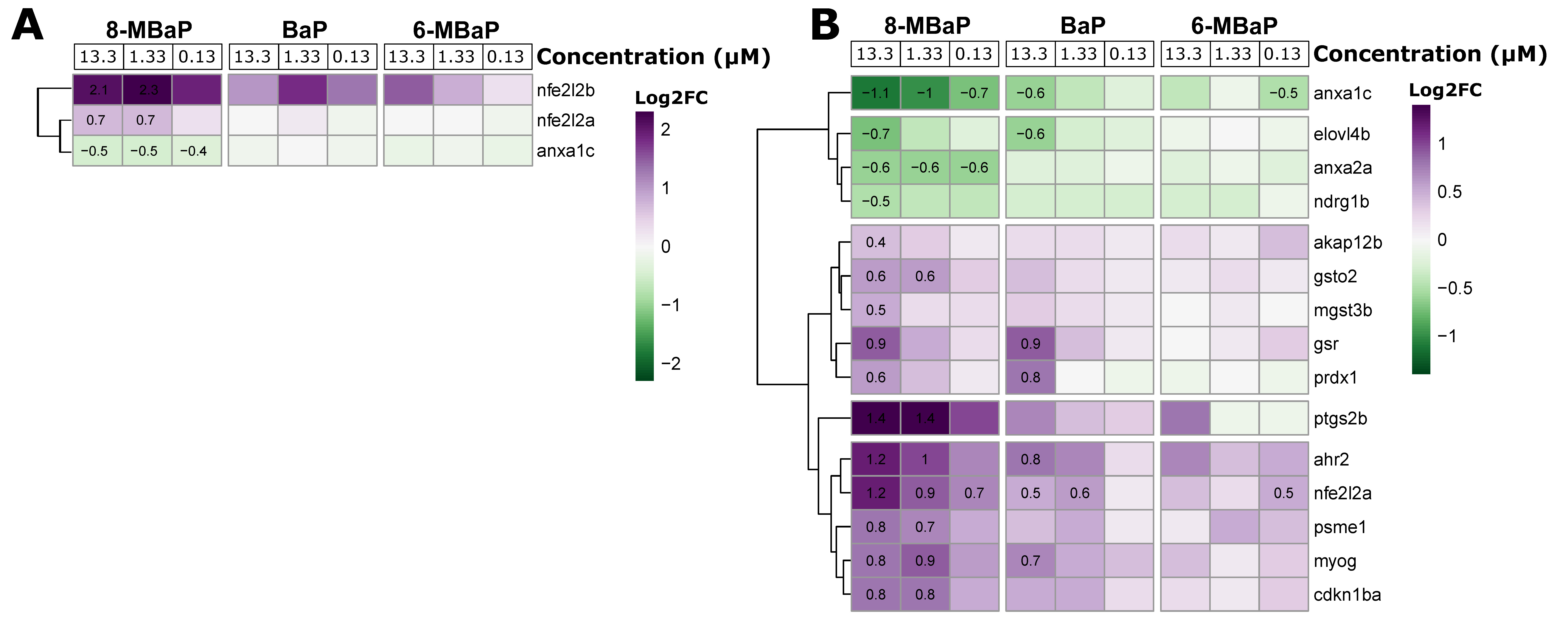


**Supplemental Figure 8:** Clustered heatmap of log_2_ fold change (log_2_FC) of genes within the oxidative stress response functional group for x-fin-associated genes at **(A)** 48 hpf and **(B)** 72 hpf for each chemical, chemical 8-methybenzo(a)pyrene (8-MBaP), 6-methybenzo(a)pyrene (6-MBaP), or benzo(a)pyrene (BaP). RNA extracted from pools of 6 whole embryos at 48 and 72 hpf, n=4.
